# Comparing the Effectiveness of Different Approaches to Raise Awareness About Antimicrobial Resistance in Farmers and Veterinarians of India

**DOI:** 10.3389/fpubh.2022.837594

**Published:** 2022-06-16

**Authors:** Garima Sharma, Florence Mutua, Ram Pratim Deka, Rajeshwari Shome, Samiran Bandyopadhyay, Bibek Ranjan Shome, Naresh Goyal Kumar, Delia Grace, Tushar Kumar Dey, Johanna Lindahl

**Affiliations:** ^1^Department of Medical Biochemistry and Microbiology, Uppsala University, Uppsala, Sweden; ^2^Department of Biosciences, International Livestock Research Institute, Nairobi, Kenya; ^3^Department of Clinical Sciences, Swedish University of Agricultural Sciences, Uppsala, Sweden; ^4^Bacteriology Lab 1, 2, National Institute of Veterinary Epidemiology and Disease Informatics, Bangalore, India; ^5^Eastern Regional Station, ICAR-Indian Veterinary Research Institute, Kolkata, India; ^6^Dairy Microbiology Division, National Dairy Research Institute, Karnal, India; ^7^Food and Markets Department, Natural Resources Institute, Chatham, United Kingdom

**Keywords:** antimicrobial resistance, dairy farmers, veterinarians, animal health, intervention, antibiotic use, one health

## Abstract

**Background:**

Antimicrobial resistance (AMR) is a global public health threat. The indiscriminate use of antibiotics in the animal health sector contributes to increasing rates of AMR and studies involving dairy farmers in India have found knowledge levels regarding antibiotics and AMR to be very low. The purpose of this study was to assess different methods to raise awareness and knowledge about AMR and antibiotic use among dairy farmers, paravets (veterinary assistants), and veterinarians.

**Materials and Methods:**

The study was conducted in September-December of 2018 in some parts of Haryana, Assam, Karnataka, and West Bengal. It had two parts: an intervention meeting (September–October 2018) which consisted of focus group discussions (FGD) with farmers, key informant interviews (KII) with veterinary professionals along with distribution of information packages, and then a follow-up survey (November–December 2018). The villages were randomly allocated to either one of the four intervention approaches (1-FGD/KII and information package on AMR; 2-FGD/KI and information on animal health; 3- FGD/KII and information package on animal health plus information on AMR; or 4- only the FGD/KII). A follow-up survey was done to assess the effect of interventions.

**Results:**

In total, 274 dairy farmers and 51 veterinary professionals (21 veterinarians and 30 paravets) participated in the follow-up survey. Many of the farmers and veterinary professionals who participated in the follow-up survey had been part of the intervention meetings. The average knowledge score of farmers was 7.8. It was found that the knowledge score was higher amongst farmers who had participated in the intervention meetings (*p* < 0.05), had received intervention approach 2 (*p* = 0.03) or approach 3 (*p* = 0.01), and amongst female farmers (*p* = 0.03) compared to male. The veterinary professionals had good knowledge but lacked interest in training the farmers about antimicrobial resistance.

**Conclusion:**

Our research demonstrated that a higher percentage of farmers and veterinary professionals who attended the intervention meeting had improved knowledge. Dairy farmers should be regularly educated on antibiotic usage and how to avoid misusing them. Also, veterinary experts should be provided with tools and strategies to educate farmers on the use of antimicrobials.

## Introduction

Antimicrobial resistance (AMR) is a global public health concern, but it is particularly serious in developing countries like India (1, 2), where the burden of infectious diseases is very high, causing the use of antibiotics to be more common and AMR elevated (3–5). AMR development and dissemination are complex problems aggravated by the expectations, interactions of prescribers and patients, as well as limited awareness, a permissive regulatory framework, and easy access to antimicrobials (6, 7). Common pathogenic bacteria in India have some of the highest antibiotic resistance rates globally (8, 9).

Antibiotics are widely used in livestock for therapeutic and prophylactic purposes, and sometimes to promote growth (10). Antibiotic usage in livestock poses a concern to human health because antibiotic-resistant bacteria can be passed from animals to humans through animal-source foods and the environment (e.g., human sewage and runoff water from agricultural sites) (11). India is a country with a large livestock population (12, 13). Antibiotics are extensively misused in the dairy sector and residues remain largely untested in milk, which is an essential part of Indian diets (8, 14, 15). Antibiotics are commonly used as growth promoters in livestock, such as poultry, however the real extent of this practice is unknown. Antibiotics such as tetracycline, doxycycline, and ciprofloxacin, which are critical to human health, are commonly used for growth promotion (8, 15). In the livestock sector, India is among the top five consumers of antibiotics (16). However, we do not really know how many antibiotics are used in the livestock industry or how much of a role they play in human antibiotic resistance (17).

There is a lack of evidence regarding the extent to which farmers are aware about the effects of antibiotic use, its residues, and resistance. Nevertheless, a few studies have revealed a poor level of knowledge among the dairy farmers about both antimicrobial residues and resistance (14, 18, 19). It has been observed that some farmers treat their animals with antibiotics regardless of whether the disease is caused by bacteria or not, and often they are not even aware that the drug is an antibiotic (14). Sick animals are usually treated with broad-spectrum antibiotics based on personal experience or through social peer learning networks, such as elders or influential farmers who have successfully treated their livestock in the past (18).

The strategies adopted by the Indian Government, including India's National Action Plan (NAP) for AMR (20) and antibiotic residue limits in food from animal origin set by Food safety and standards authority of India (FSSAI) (21), try to address critical policy and regulatory challenges surrounding antibiotic usage in accordance with One Health principles. However, implementation has been slow, and stakeholder participation is suboptimal (22). Controlling antibiotic use, monitoring resistance, and creating innovative techniques to prevent AMR in pathogens are all important aspects of addressing the problem of AMR. Raising awareness and implementing communication programs for the general public and other stakeholders involved in antibiotic use is one of the six strategic priorities of NAP, for the period 2017–2021, to tackle AMR in the country (20, 22).

Rational use of antibiotics by veterinarians is one of the critical cornerstones of prevention and control of AMR in the livestock sector. Antimicrobial usage and its possible link to public health should be primary focus of attention for all stakeholders, including dairy farmers. However, little research has been done to examine the behavior of veterinarians and dairy farmers of India when it comes to prescribing and administering antimicrobials. Poor availability of customized training material for providing training on AMR is also considered as an important limitation in the country (17). There is also a scarcity of baseline data, which is essential to determine the current situation (23).

In this context, a study was conducted to assess the effectiveness of delivering training to dairy farmers, paravets and veterinarians in terms of improving awareness. Different approaches of raising awareness were tested, and the change in knowledge in regard to use of antimicrobials and AMR was assessed after the intervention.

## Materials and Methods

### Study Areas

**Figure 1:**
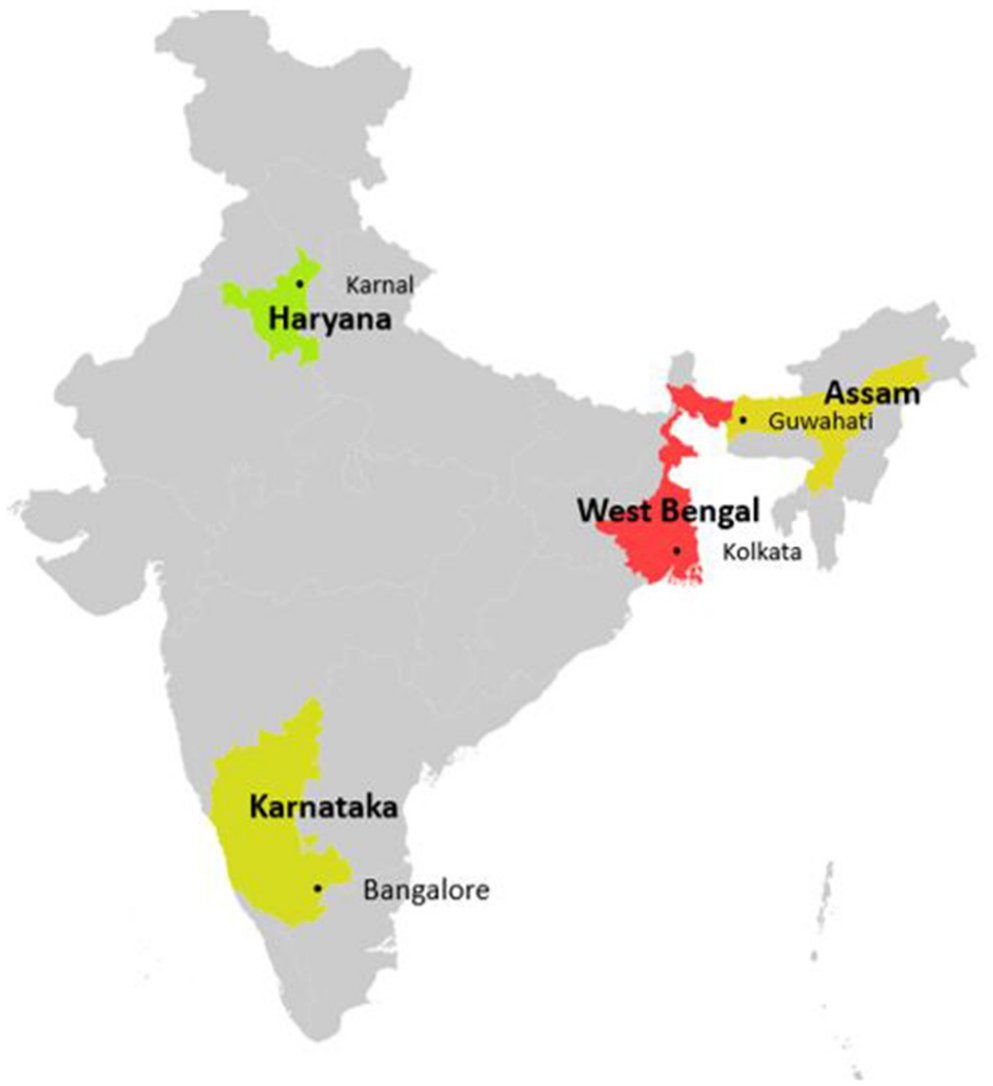
Indian map highlighting the states where the study was conducted.

The study was conducted in 2018, during the months of September-December. The study covered urban and peri-urban areas of four cities in India viz. Guwahati (in Assam), Karnal (in Haryana), Bangalore (in Karnataka) and Kolkata (in West Bengal) that are located in different parts of the country (Figure 1). In the cities of Guwahati, Karnal and Bangalore, a total of eight wards/villages (4 urban and 4 peri-urban/rural) were included while in Kolkata, only four wards/villages were selected (2 peri-urban/rural and 2 urban) due to the larger distance between the villages and the district headquarters. Study wards/villages in Karnal and Guwahati participated in our previous studies where they had been randomly selected (24, 25). In Bangalore and Kolkata, study wards/villages were randomly selected from a list of wards/villages near the district headquarters.

The stakeholders included in the study were dairy farmers, veterinarians and paravets. Farmers who owned at least one milking cow or buffalo were counted as dairy farmers; the veterinarians were the veterinary doctors who held a university veterinary degree and were responsible for the treatment of diseased animals in the respective villages, and paravets were a category of animal health workers who did not have a university veterinary degree but had a diploma on veterinary services or had received training from different sources on veterinary first aid services and provided relevant services to the dairy farmers in the study areas (26).

### Study Design

The study was conducted in two phases:

Phase -I: Intervention part (September-October 2018).

Phase-II: Follow-up survey (November-December 2018).

#### Intervention Part

The villages were randomly allocated to either one of four intervention approaches. In Guwahati, Karnal, Bangalore where eight villages were included, there were 2 villages (one rural and one urban) for each intervention group, however, in case of Kolkata, only one village was considered for each intervention. The type of interventions included the following:

- **Conventional AMR approach:** Under this approach, relevant stakeholders in the village/ ward received information specific to AMR including prudent use of antibiotics, drug withdrawal periods etc.
- **Animal health approach:** Under this approach, the stakeholders received messages on animal health and productivity (biosecurity, diseases and their control, available vaccination options etc.).
- **Animal health and simplified conventional AMR approach:** Under this approach, the stakeholders received same messages as the animal health category (2) as well as simplified AMR messages.
- **Just discussions**: Under this approach, the stakeholders were a part of focus group discussions (FGD) and key informant interviews (KII) but received no training or any intervention.

In September-October 2018 during the intervention meetings, discussions focusing primarily upon animal health and AMR were conducted with the dairy farmers, using a semi-structured focus group discussion guide and a key informant interview guide for the paravets, veterinarians (Supplementary Materials 1, 2). The results of the FGDs and KIIs have been summarized elsewhere (19). After this activity, extension materials developed in English (Supplementary Material 3), translated into local languages, were distributed as a part of the intervention in each ward/village along with a brief (10–15 min) explanation to the participants. Different extension material was given to villages in intervention group 1, 2, and 3, and no material was given to villages where just discussions were done i.e., the 4^th^ intervention group.

**Figure 2:**
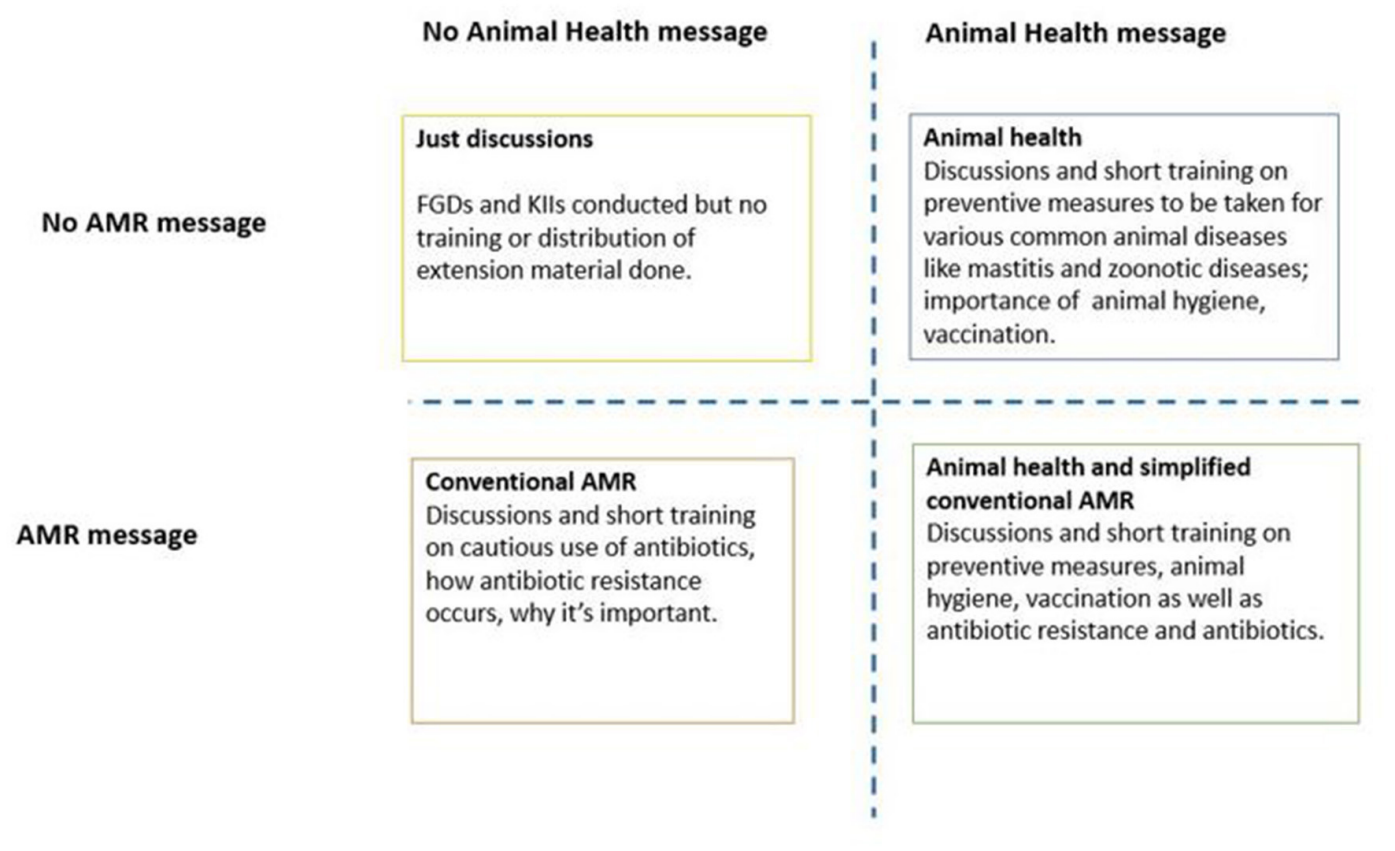
Explains various intervention approaches.

As summarized in Figure 2, and depending on the type of the intervention, the participants were made aware on animal health and disease control including biosecurity (group 2 and 3), antibiotic use in dairy animals (group 1 and 3), antibiotic residues in animal-source foods as well as the risk of AMR in humans (group 1 and 3). The disease component (group 2 and 3) focused on mastitis, brucellosis, leptospirosis, and Q fever (their causes, manifestation, impact, and control). The participants were provided with take home materials which included pamphlets and posters.

#### Follow-Up Survey

In November and December of 2018, a follow-up survey was conducted to evaluate the efficacy of the interventions. Two surveys were designed, one for dairy farmers (Supplementary Material 4) and the other for veterinarians and paravets (Supplementary Material 5). These questionnaires were piloted, revised based on feedback, finalized, and used to collect the necessary data.

Data were collected from the same villages. Any farmer, veterinarian and paravet was allowed to participate, including both those who had previously participated in the discussions (FGD and KII) as well as some who had not participated. The questionnaire for the dairy farmers was divided into seven sections and that for the professionals was divided into five sections. Both open-ended and closed-ended questions were included. The questions were about the socio-demographic characteristics of the participants, their knowledge about antibiotics, zoonotic diseases, AMR, beliefs of the participants, perceived risk, and some attitude questions on antibiotics, antibiotic resistance.

### Data Analysis

The data was entered in Microsoft Excel and double-checked for missing entries, typing and other errors. Stata/SE 15.1 software (STATACorp Ltd, College Station, Texas, USA) was used for data analysis. Each farmer was given a score based on how well they answered questions related to antibiotic use and AMR. A score of “1” was given for each correct answer to each question, and a score of “0” was given for incorrect answers.

The dairy farmers with <10 milking cows/buffaloes were designated as small scale and those with 10 or more than 10 milking cows/buffaloes were deemed large scale (Table 1). Descriptive statistics for the farmer data was performed by calculating proportions and frequencies to describe the demographic characteristics and knowledge, attitude, and practice-related variables. A Chi-square test was used to check for associations between knowledge indicators on antibiotics, AMR, takeaways from the intervention survey discussions and the type of intervention approach.

**Table 1:** Showing the sociodemographic factors of the dairy farmers across various study sites.

	**Karnal (*N* = 81)**	**Guwahati (*N* = 78)**	**Bangalore (*N* = 75)**	**Kolkata (*N* = 40)**	**Total (*N* = 274)**
Females	12 (14.8%)	10 (12.2%)	15 (20%)	14 (35%)	51 (18.61%)
Males	69 (85.2%)	68 (87.2%)	60 (80%)	26 (65%)	223 (81.38%)
Small-scale dairy					
farmers*	74 (91.4%)	33 (42.3%)	75 (100%)	40 (95%)	222 (81.02%)
Large-scale dairy farmers*	7 (8.6%)	43 (55.1%)	0 (0%)	2 (5%)	52 (18.97%)
**Education**					
No education	45 (55.6%)	33 (42.3%)	18 (24%)	13(32.5%)	109 (39.78%)
Till 10^th^ standard	28 (34.6%)	23 (29.5%)	37 (49.3%)	14 (35 %)	102 (37.22%)
Senior secondary (12^th^					
standard)	3 (3.7%)	9 (11.54%)	15 (20%)	6 (15%)	33 (12%)
Graduate/diploma holder	5 (6.2%)	13 (16.7%)	5 (6.7%)	7 (17.5%)	30 (10.94%)
**Age**					
20–40 yrs.	24 (29.6%)	26 (33.3%)	15 (20%)	9 (22.5%)	74(27%)
41–60 yrs.	47 (58%)	44 (56.4%)	44 (58.7%)	25 (62.5%)	160(58.39%)
Above 60 yrs.	10 (12.4%)	8 (10.3%)	16 (21.3%)	6 (15%)	40(14.59%)

*Small-scale dairy farm was defined as <10 milking cows/buffaloes, while large-scale had more than or equal to 10.

Cronbach's alpha score was used to assess internal consistency within the knowledge indicators. First a univariate linear regression was done to check the association between our continuous outcome-knowledge score and type of intervention approach, participation in the FGDs, independent variables like gender and education status. The variables with a *p* < 0.05 were selected for the multivariable linear regression model. Multivariable linear regression was done to assess the association between the knowledge score of the farmers and the independent variables type of intervention approach, participation in the FGDs, and gender. For the veterinarian and paravet data, only descriptive statistics were performed. Bivariate analysis/ approach wise classification was done for the veterinary professionals (veterinarians and the paravets) related to their perspective about AMR, about how they will explain AMR to the dairy farmers and things remembered by them from the KII discussions. A *p* < 0.05 was considered statistically significant.

## Results

### Dairy Farmers Follow Up Survey

A total of 274 farmers were interviewed in the follow-up survey. Most of the interviewed farmers were males (81%) and were aged between 41 and 60 years (58 %) (Table 1). Out of the total farmers interviewed in the follow-up survey, 71 (25.9%) were in intervention 1 (conventional AMR), 72 (26.3%) in intervention 2 (animal health), 67 (24.5%) in intervention 3 (animal health and simplified conventional AMR), and 64 (23.4%) in intervention 4 (just discussions), with an even participation in each of the four states (Table 2).

**Table 2:** Total number of dairy farmers participating in different intervention approaches in various study sites.

**Intervention approach**	**Study sites**			
	**Karnal (*N* = 81)**	**Guwahati (*N* = 78)**	**Bangalore (*N* = 75)**	**Kolkata (*N* = 40)**
Conventional AMR	22 (27.2%)	20 (25.6%)	18 (24%)	11 (27.5%)
Animal health	20 (24.7%)	20 (25.6%)	22 (29.3%)	10 (25%)
Animal health and simplified conventional AMR	20 (24.7%)	20 (25.6%)	18 (24%)	9 (22.5%)
Just discussions	19 (23.5%)	18 (23.1%)	17 (22.7%)	10 (25%)

#### Farmers' Takeaways From Focus Group Discussions

**Figure 3:**
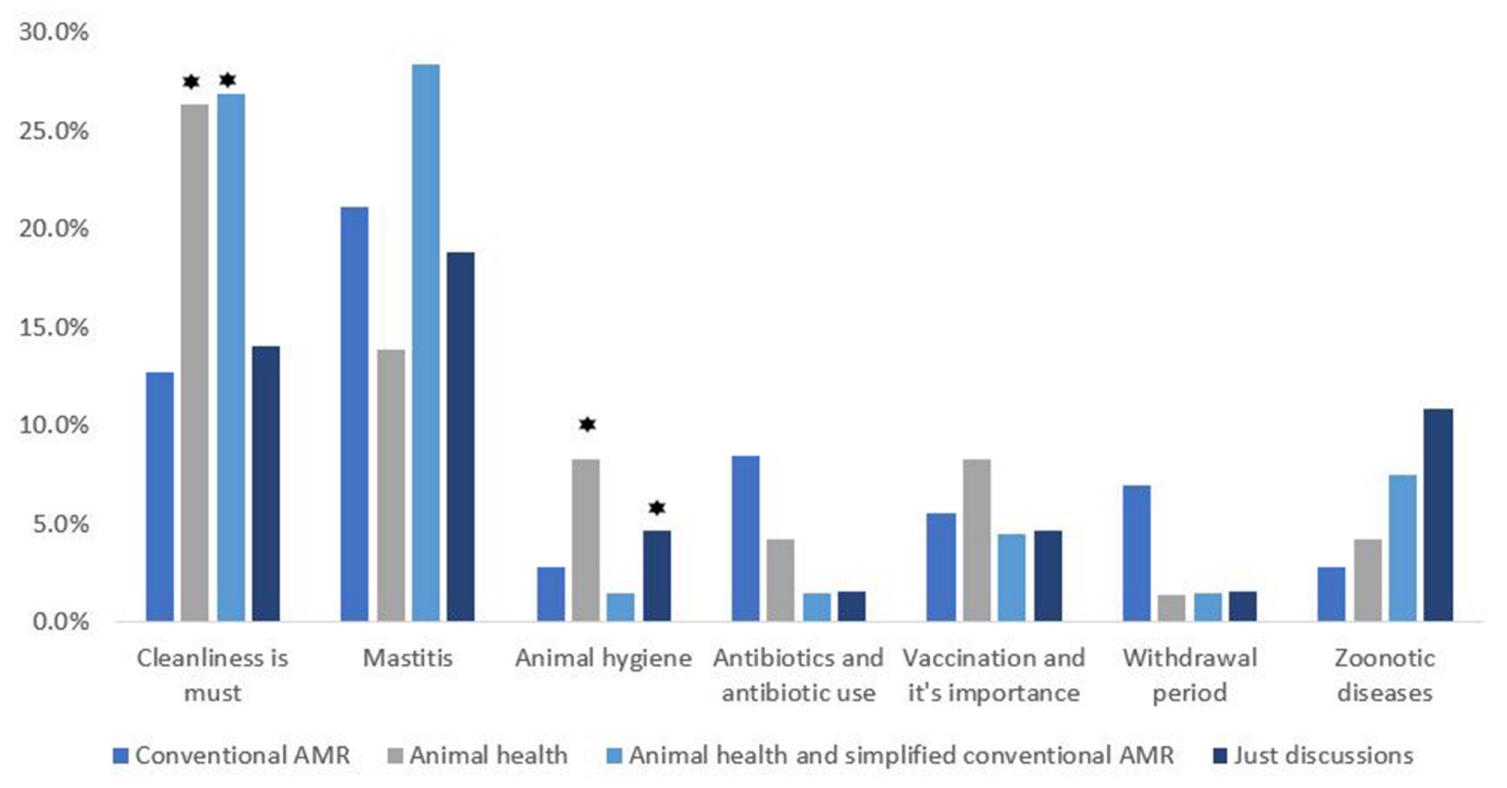
Intervention wise classification of the topics remembered by the dairy farmers from FGDs conducted in the intervention part (⋆ highlights the approaches with a significant difference from the others).

Out of 274 dairy farmers, 190 (69.3%) farmers said that they had participated in the focus group discussions conducted in the intervention part. The most common topics that dairy farmers remembered from the FGDs are mentioned in Figure 3 and Table 3.

**Table 3:** Intervention wise classification of topics remembered by farmers on aspects on management of diseased animals.

**Intervention approach**	**Variables related to management of diseased animals**	
	**Consult a veterinarian for sick animals**	**Separate sick from health animals**
Conventional AMR	1 (1.4%)	1 (1.4%)
Animal health	0	4 (5.6%*)
Animal health and simplified conventional AMR	3 (4.5%)	6 (9%*)
Just discussions	4 (6.3%)	0

*The approaches with an asterisk were significantly different from the others.

#### Referral Pattern and Knowledge on Antibiotics and AMR

We found that majority of the farmers (79.9%; *n* = 274) reported to have called a veterinary doctor or paravet on their farm whenever they had a sick animal, 14.2% treated the sick animal on their own, 4% took the sick animals to the government veterinary hospital, 1.1% sought help from neighbors, and 0.7% sought advice from the local pharmacist. Consultation costs varied; less than 500 Indian rupees (INR) (6.57 USD) (78%), 500–1,000 INR (6.57–13.15 USD) (15%), and more than 1,000 INR (13.15 USD) (7%). Three farmers said the consultation fee was dependent on the case and varied from veterinarian to veterinarian.

**Figure 4:**
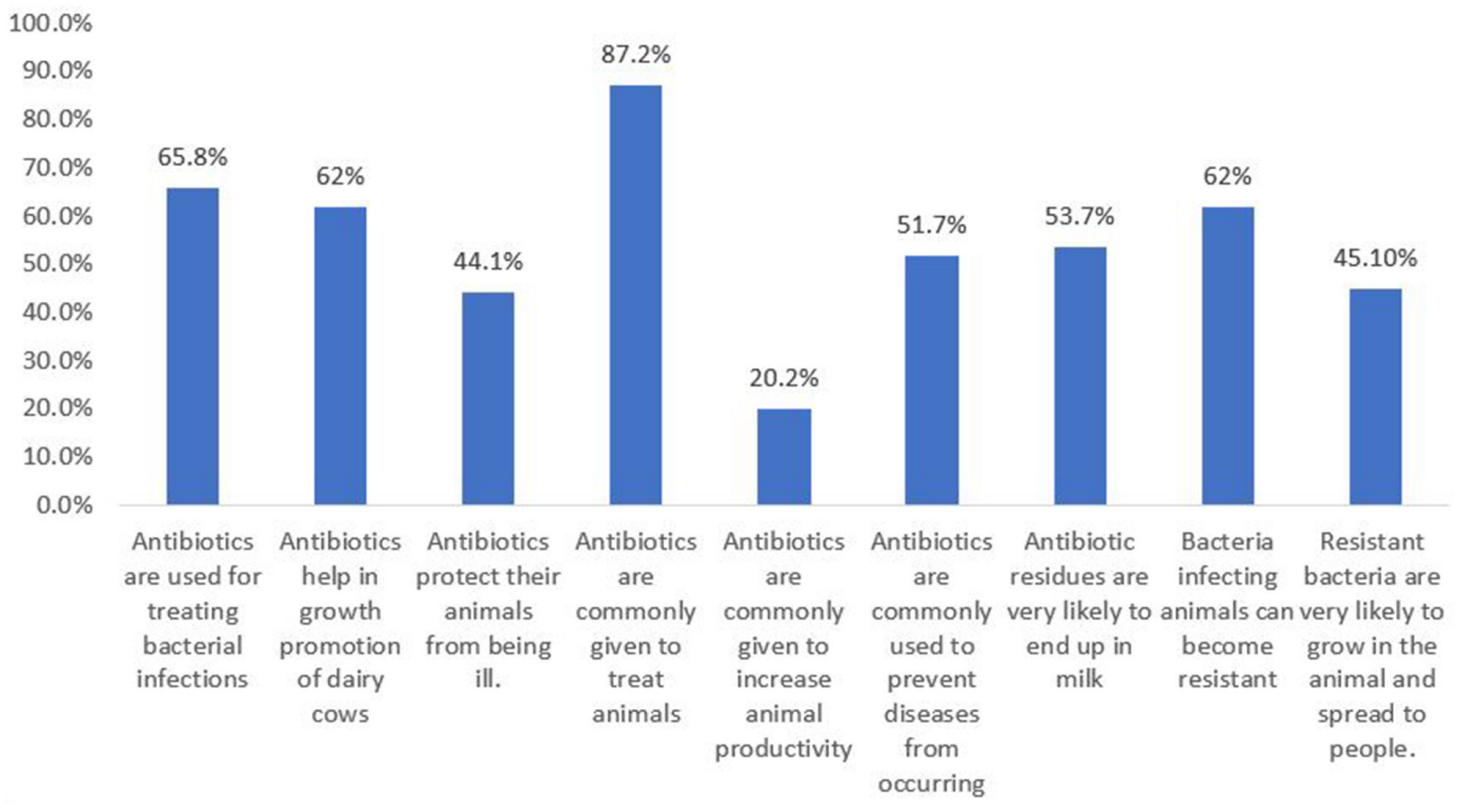
Total percentage of the dairy farmers responding to the knowledge variables related to antibiotics and AMR.

Out of 272 (2 no responses-NR), 179 (65.8%) dairy farmers responded that antibiotics are used for treating bacterial infections; according to 62 (22.8%) of the 272 dairy farmers antibiotics help in growth promotion of dairy cows; 120 (44.1 %) out of 272 believed that antibiotics protect their animals from being ill. Antibiotics are commonly given to treat animals, according to 238 (87.2%) out of 273 (1 NR) dairy farmers; antibiotics are commonly given to increase animal productivity, according to 55 (20.2%) out of 273 (1 NR) dairy farmers; and antibiotics are commonly used to prevent diseases from occurring, according to 141 (51.7 %) of 273 (1 NR) dairy farmers (Figure 4).

Out of 274 dairy farmers, 147 (53.7%) believed that antibiotic residues are very likely to end up in milk. Sixty two percent (*n* = 273) (1 NR) agreed that bacteria infecting animals can become resistant; 123 (45.1%) out of 273 (1 NR) dairy farmers agreed that resistant bacteria are very likely to grow in the animal and spread to people (Figure 4).

Bivariate analysis of the dependent variable intervention approach with the independent knowledge variables on antibiotics and AMR revealed that none of the knowledge variables were significantly associated with the type of intervention approach (*p* > 0.05) (Table 4).

**Table 4:** Shows the intervention wise classification of responses to knowledge questions on antibiotics and AMR.

	**All farmers**	**Classic AMR** **(*N* = 71)**	**Animal health** **(*N* = 72)**	**Animal health and simplified conventional AMR (*N* = 67)**	**Just discussions (*N* = 64)**	***P*-value**
Antibiotics are for treating bacterial infections	179/272 (65.8%)	42 (60%)	42 (58.3%)	47 (71.2%)	48 (75%)	0.10
Antibiotics help animals grow better	62/272 (22.8%)	21 (30%)	10 (13.9)%	18 (27.3%)	13 (20.3%)	0.09
Antibiotics prevent the animals from getting sick	120/272 (44.1%)	27 (38.6%)	35 (48.6%)	31 (46.1%)	27 (22.5%)	0.28
Antibiotics are given to treat diseases	238/273 (87.2%)	65 (92.9%)	59 (81.9%)	59 (88.1%)	55 (85.9%)	0.27
Antibiotics are given to increase the productivity in dairy animals	55/273 (20.1%)	16 (22.9%)	12 (16.7%)	17 (25.4%)	10 (15.6%)	0.42
Antibiotics are given to stop the diseases from happening	141/273 (51.6%)	43 (61.4%)	31 (43.1%)	32 (47.8%)	35 (54.7%)	0.14
Some antibiotic residues can get into the milk	147/274 (53.6%)	35 (49.3%)	41 (56.9%)	38 (56.7%)	33 (51.6%)	0.62
The germs infecting the animal can become resistant	170/273 (62.3%)	41 (57.7%)	46 (64.8%)	41 (61.2%)	42 (65.6%)	0.90
Resistant bacteria can develop in the animal, and transfer to the human	123/273 (45%)	35 (49.3%)	33 (46.5%)	32 (47.8%)	23 (35.9%)	0.55

#### Zoonotic Diseases: Knowledge and Behavior

**Figure 5:**
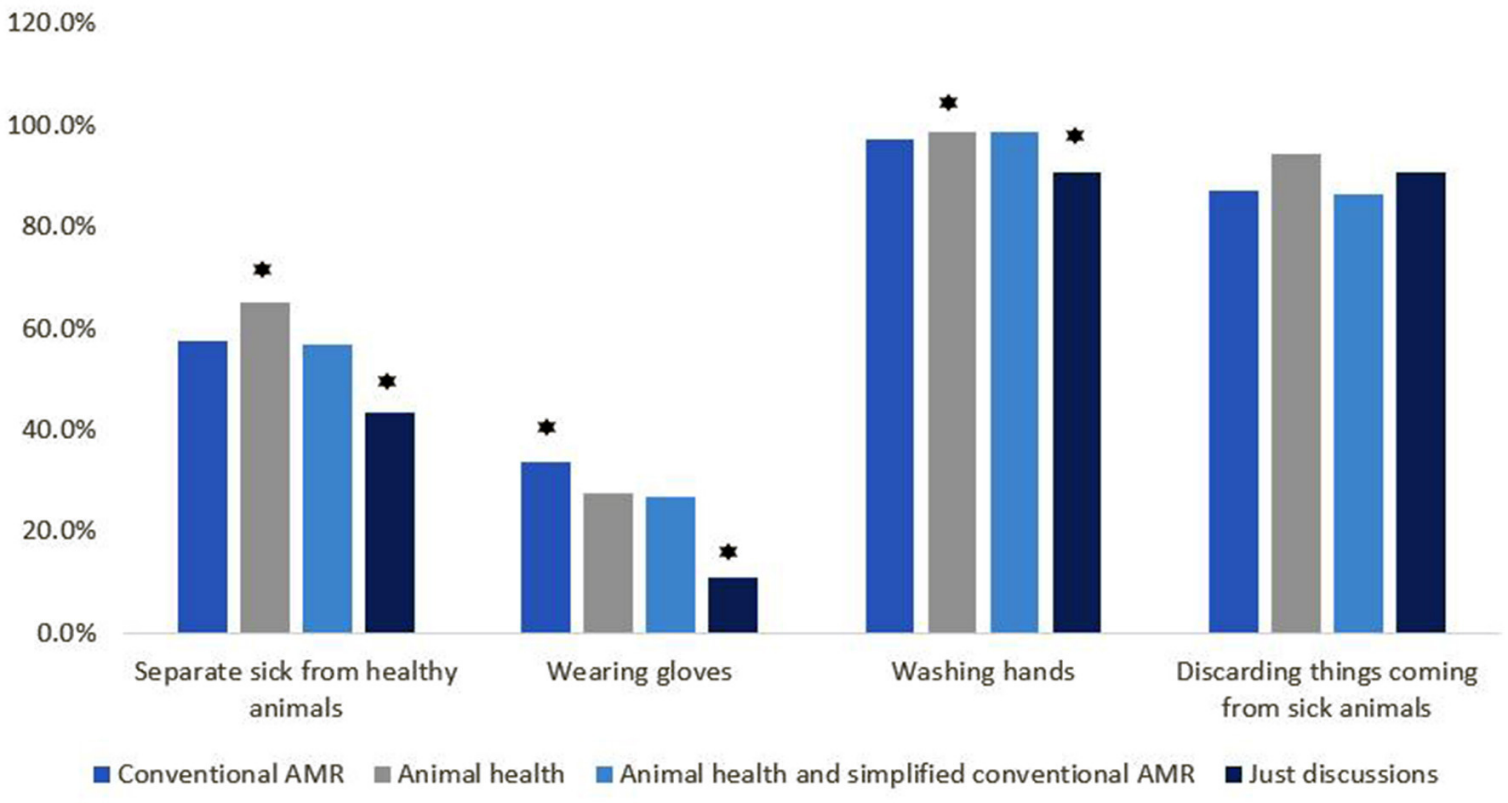
Intervention wise classification of the knowledge variables on preventive measures to take to avoid infections related to zoonotic diseases (⋆ highlights the approaches with significant difference).

Out of 273 dairy farmers, 128 (47%) were aware that animals can transmit diseases to humans. When asked to name such a disease, 25 (9.1 %) of the 274 farmers mentioned rabies, 17 (6.2%) mentioned brucellosis, and 3 (1.1%) mentioned anthrax. Fourteen (5.1%) mentioned foot and mouth disease (FMD), which is not a zoonosis. Regarding the mode of transmission of infection from sick animals to humans, direct contact was mentioned by 119 (43.6%) of 273 respondents (1 NR); inhalation by 124 (45.4%); ingestion was the mode of transmission as per 94 (34.4%); and contact with animal products was mentioned by 152 (55.7%). When handling a sick animal, 154 (56.2%) out of 274 respondents mentioned separating sick animals from healthy ones as a preventative and control measure; 69 (25.2%) said wearing gloves is important; 264 (96.4%) respondents said washing hands is very important; and 246 (89.8%) mentioned discarding everything that comes from a sick animal as a preventative and control measure (Figure 5 and Table 5).

**Table 5:** Intervention wise classification of the dairy farmers on various knowledge indicators related to zoonotic diseases.

		**Conventional AMR (*N* = 70)**	**Animal health** **(*N* = 72)**	**Animal health and simplified conventional AMR (*N* = 67)**	**Just discussions (*N* = 64)**
Aware of diseases people get from sick animals		33 (47.1%)	35 (48.6%)	35 (52.2%)	25 (39.1%)
Mode of transmission	Contact	35 (50%)	23 (31.9%)	34 (50.7%)	27 (42.1) %
	Inhalation	35 (50%)	28 (38.9%)	31 (46.3%)	30 (46.8%)
	Ingestion	22 (31.4%)	27 (37.5%)	24 (35.8%)	21 (32.8%)
	Contact with animal products	40 (57.1%)	44 (61%)	40 (59.7%)	28 (43.7%)

#### Perceived Risk

Ninety-one (91; 33.2%) out of 274 dairy farmers were not at all worried about getting any infection from livestock; 86 (31.4%) were a little bit worried and 97 (35.4%) were actually worried. One-eighty-nine (69%) said it was impossible or very unlikely to get infected with a resistant bacterium while visiting a hospital; and 63 (23%) said there was a high probability of getting infected with a resistant bacteria while visiting a hospital; and 22 (8%) stated that they did not know. Out of 274, 165 dairy farmers (60.2%) said there was no chance or a very small chance they would get infected with resistant bacteria from livestock; 67 (24.5%) said there's a good chance they would get infected with resistant bacteria from livestock; and 42 (15.3 %) stated they have no idea. Out of 274 respondents, 188 (68.6%) thought the problem of resistant bacteria was fairly serious; 36 (13.1%) thought it was a minor issue; 11 (4%) said it was not a concern for them; and 39 (14.2%) said they didn't know.

Majority of the respondents (231; 84.3%) strongly agreed that it is always advisable to call a veterinarian so that the animal receives the proper treatment. The veterinarian delivers the best advice, according to 241 (87.9 %). Antibiotics should not be used in excess, according to 191 (69.7 %), as this can lead to even more significant difficulties in the future. Out of 274 respondents 95 (34.7%) agreed to the statement that they cannot throw away the milk while the animal is undergoing treatment; they need to sell it for money, and they do not think it is all that harmful to consume it.

Bivariate analysis was done to see if the statements mentioned above are associated with the type of intervention approach and it was found that there was no significant association (p>0.05) Table 6.

**Table 6:** Shows the number of farmers agreeing to different statements, based on the training they had received.

	**All farmers**	**Conventional AMR**	**Animal health**	**Animal health and simplified conventional AMR**	**Just discussions**	***P* value**
1. It is always advisable to call a veterinarian so that the animal receives proper treatment	231 (84.3%)	57 (80.3%)	61(84.7%)	57(85.1%)	56(87.5%)	0.23
2. The veterinarian delivers the best advice	241 (88.3%)	61 (85.9%)	59 (81.9%)	61 (91%)	60 (95.2%)	0.09
3. Antibiotics should not be used in excess as this can lead to significant difficulties in the future	191 (69.7%)	51 (71.8%)	50 (69.4%)	44 (65.7%)	46 (71.9%)	0.78
4. Can't throw away the milk when the animal is on treatment. It's not that harmful to consume it Anyway	95 (34.7%)	17 (23.9%)	25 (34.7%)	28 (41.8%)	25 (39.1%)	0.56

#### Cronbach's Alpha

Cronbach's alpha was used to measure the internal consistency of the variables in the scale, and it is expressed as a number between 0 and 1 (27). There were a total of 22 knowledge variables in the knowledge scale, and when optimized for Cronbach's alpha score, 8 variables showed acceptable consistency, 0.78, showing consistency within these knowledge variables.

#### Multivariable Analysis

A knowledge score was calculated for all the dairy farmers by calculating the sum of correct answers. The average score was 7.8 (median 7, range 13). It was found that the knowledge score of the farmers was higher in the ones who had previously participated in focus group discussions (*p* < 0.05), received intervention in approach 2-animal health (*p* = 0.03), approach 3-animal health and AMR (*p* = 0.01) and female dairy farmers (*p* = 0.03) (Table 7).

**Table 7:** Multivariable regression results with participation in FGDs, Intervention approach and gender.

	Coefficient*	**Standard error**	***P*-value**	**95% CI (confidence interval)**
**Participated in FGD**				
Yes	Reference			
No	−1.73	0.41	<0.001	−2.55, −0.90
**Approach**				
Just discussions	Reference			
Conventional AMR	0.96	0.55	0.08	−0.13, 2.06
Animal health	1.17	0.55	0.03	0.06, 2.27
Animal health and simplified conventional AMR	1.47	0.58	0.01	0.33, 2.62
**Gender**				
Male	Reference			
Female	1.08	0.51	0.03	0.07, 2.10
_cons	7.24	0.44	0.00	6.36, 8.12

*Change in knowledge score.

### Follow-Up Survey Results for the Veterinary Professionals

A total of 51 veterinary professionals (21 veterinarians and 30 paravets) participated in the follow up survey. Majority were males (90.2%) and aged between 41 and 60 yrs. (52%) (Table 8). Out of these 51 professionals, 14 (27.5%) were a part of conventional AMR approach in the intervention meetings, 13 (25.5%) were a part of animal health approach, 12 (23.5%) were a part of animal health & simplified conventional AMR approach, and 12 (23.5%) were a part of just discussions. Out of 51, 43 (91.5%) had participated in the KII discussions (Table 9) mentions the number of veterinary professionals participating in different intervention approaches across the study sites.

**Table 8:** Distribution of sociodemographic factors of the veterinarians in each state.

	**Karnal (*n* = 16)**	**Guwahati (*n* = 8)**	**Bangalore (*n* = 17)**	**Kolkata (*n* = 10)**
Veterinarians	7 (43.8%)	4 (50%)	8 (47.1%)	2 (20%)
Paravets	9 (56.3%)	4 (50%)	9 (52.9%)	8 (80%)
Male	16 (100%)	8 (100%)	16 (94.1%)	6 (60%)
Female	0	0	1 (5.9%)	4 (40%)
20–40 yrs.	9 (56.3%)	1 (12.5%)	5 (29.4%)	6 (60%)
41–60 yrs.	4 (25%)	7 (87.5%)	12 (70.6%)	4 (40%)
Above 60 yrs.	3 (18.8%)	0	0	0

**Table 9:** Number of veterinary professionals participating in different intervention approaches in various study sites.

**Intervention Approach**	**Karnal (*N* = 81)**	**Guwahati (*N* = 78)**	**Bangalore (*N* = 75)**	**Kolkata (*N* = 40)**
Conventional AMR	4 (25%)	3 (37.5%)	4 (23.5%)	3 (30%)
Animal health	4 (25%)	1 (12.5%)	5 (29.4%)	3 (30%)
Animal health and simplified conventional AMR	4 (25%)	2 (25%)	4 (23.5%)	2 (20%)
Just discussions	4 (25%)	2 (25%)	4 (23.5%)	2 (20%)

#### Knowledge and Practices Related to Antibiotics, AMR

Twenty-nine (56.9%) out of 51 veterinary professionals had prescribed antibiotics in the last two reported cases before the interview. When asked how they would explain antibiotics to the farmers, 22 (43.1%) out of 51 responded that they will just say that antibiotics treat diseases; 12 (23.5%) responded they will say that antibiotics are used to kill bacteria; 3 (5.9%) out of 51 said they will say that antibiotics are given in case of fever or mastitis; 4 (7.8%) of them responded they will just ask the farmers to always consult a veterinarian before giving any medicine, and if they are asked to follow a withdrawal period, they should do it.

Antibiotic resistance occurs when antibiotics stop functioning due to overuse, according to 34 (66.7%) out of 51 veterinarians and paravets. Antibiotic resistance occurs when the course of antibiotics is not completed, according to 8 (15.7 %) of the 51 survey respondents. 4 (7.8%) said resistance occurs when antibiotics cease working because the pathogen has evolved; 3 (5.9%) said resistance occurs when low-generation antibiotics stop working due to the usage of high-generation antibiotics.

When asked how they would explain AMR to the farmers, 23 (45.1%) said that they will just ask the farmers to consult a veterinarian before giving any medicine to the animals, complete the course, follow withdrawal if asked to and not to overuse; 21 (41.2%) out of 51 said that the farmers won't understand and there is no need of explaining it to them; 4 (7.8%) said that they will tell them that if one medicine stops working, another medicine needs to be given and 5 (9.8%) of them said that they will ask for a laboratory diagnosis and why is it important before giving any antibiotic.

#### Perspective of the Veterinarians Related to Antibiotics and AMR

Veterinarians and paravets were given two scenarios in which they had to converse with two imaginary colleagues and were asked who they agreed with. The first scenario was where “*colleague 1 says: It is important to give the farmer antibiotics when he has a sick animal, otherwise he will not be happy and not call me again and colleague 2 says: I think we should not give farmers so many antibiotics, only if we are really sure it is a bacteria causing the disease”*. 39 (76.47%) out of 51 surveyed veterinarians and paravets agreed with colleague 2 and 3 (5.88%) agreed with colleague 1.

The second scenario was where “*colleague 1 says: I always tell the farmers that he must throw away the milk during medicine treatment and 2 days after, otherwise the milk can be harmful and colleague 2 says: Farmers cannot afford to throw away milk during treatment, so I don't even tell them to do it.”* 23 (45.1%) out of 51 strongly agreed with colleague 1, while 11 (21.6%) out of 51 agreed with colleague 2.

Thirty-nine (76.5%) out of total surveyed veterinarians said that they are worried about them or someone in the family getting an infection with a resistant bacterium. 15 (29.4%) out of 51 said that it is very likely that they are exposed to a resistant bacterium when they visit a dairy farm; 14 (27.5%) said that it is very likely that they are exposed to resistant bacteria when visiting a hospital.

The biggest issue in combating AMR, according to 14 (27.5%) of 51 veterinarians, is lack of awareness among farmers; farmers giving antibiotics to animals without consultation is the main challenge, according to 11 (21.6%) of the total. Quacks (a person who pretends to possess the skill, knowledge, or qualifications he or she does not hold) administering antibiotics to dairy animals was identified by 8 (15.7%) of them as a major concern in combating AMR. Incomplete course was also identified as a challenge in tackling AMR by 7 (13.7%) of the total veterinary professionals surveyed.

Bivariate analysis was done to check the association between various perspective variables of veterinarians related to AMR and the type of intervention approach, but no significant association was found (*p* > 0.05) (Table 10).

**Table 10:** Shows the intervention wise classification of perspective of veterinarians related to AMR.

	**All**	**Conventional AMR**	**Animal health**	**Animal health and simplified conventional AMR**	**Just discussions**	***P* value**
(1) AMR: Antibiotics stop working due to overuse	34 (66.7%)	7 (50%)	10 (76.9%)	9 (75%)	8 (66.7%)	0.43
(2) AMR: Antibiotics stop working because the course is not completed	8 (15.7%)	4 (28.6%)	1 (7.7%)	1 (8.3%)	2 (16.7%)	0.41
(3) AMR: Low generation antibiotics stop working due to use of high generation antibiotics	3 (5.9%)	2 (14.3%)	1 (7.7%)	0	0	0.33
(4) AMR: Antibiotics stop working because the pathogen gets modified	4 (7.8%)	1 (7.1%)	1 (7.7%)	1 (8.3%)	1 (8.3%)	0.99
(5) Worried about you or someone in the family may get an infection with a resistant bacteria	39 (76.5%)	12 (85.7%)	10 (76.9%)	7 (58.3%)	10 (83.3%)	0.36
(6) Think that they are exposed to resistant bacteria when they visit a dairy farm	15 (29.4%)	4 (28.6%)	5 (38.5%)	2 (16.7%)	4 (33.3%)	0.35
(7) Think it is very likely that they are exposed to resistant bacteria when you visit a hospital because most hospitals have it	14 (27.5%)	5 (35.7%)	3 (23.1%)	4 (33.3%)	2 (16.7%)	0.75
(8) Main challenge in tackling AMR: lack of awareness amongst farmers	14 (27.5%)	3 (21.4%)	2 (15.3%)	5 (41.7%)	4 (33.3%)	0.45
(9) Main challenge in tackling AMR: farmers medicating animals on their own	11 (21.6%)	2 (14.3%)	3 (23.1%)	1 (8.3%)	5 (41.7%)	0.20
(10) Main challenge in tackling AMR: quacks giving antibiotics to the farmers	8 (15.7%)	3 (21.4%)	3 (23.1%)	1 (8.3%)	1 (8.3%)	0.60
(11) Main challenge in tackling AMR: Incomplete course	7 (13.7%)	2 (14.3%)	1 (7.7%)	1 (8.3%)	3 (25%)	0.57

## Discussion

The purpose of this study was to find out if various short training interventions, relating to antibiotic, AMR, and animal health, can impact the levels of knowledge in dairy farmers, veterinarians, and paravets. Four intervention approaches were tested – one where the participant received conventional AMR messages, along with some discussion on anti-microbial resistance and antibiotics; the second one where the participants received information regarding animal health and common diseases like mastitis, zoonotic diseases, prevention measures etc.; the third one where the participants received information about both AMR and animal health; and a fourth where just focus group discussions were conducted with the farmers, and interviews were done with the paravets and veterinarians, to understand the knowledge and practices related to antibiotics, common animal diseases in the area, perspective related to AMR etc. The participants in the fourth group received no training.

No matter what the intervention was, the main things that the dairy farmers remembered from the discussions were mastitis: prevention and precautions, cleanliness, and hygiene. Mastitis was the main animal health issue that was pointed out by the dairy farmers during the discussions in the FGDs a well (19). Subclinical mastitis is a major problem in India, with rates ranging from 10 to 50 % in cows and 5 to 20 % in buffaloes, resulting in lower production and significant financial losses for dairy farmers (28, 29). Mastitis infection can be reduced by following hygienic procedures while milking, maintaining the cow, and cleanliness in the farm. Milk is produced by a huge number of smallholder dairy farmers in India, however information on milk-borne zoonosis and milk hygiene procedures is limited. There is a significant knowledge gap in terms of bovine mastitis awareness and hygiene practices (30). Our findings clearly demonstrate that when farmers are provided with information about this, there is a change in their level of understanding about the subject, likely because the issue is of great interest to them.

Many farmers also remembered having a conversation about antibiotic use, withdrawal period, and other related topics. During the FGDs, farmers were unaware of the term “antibiotic” itself, used old prescriptions to buy medicines from the pharmacy and were not aware of the health risks associated with the use of medicines in the treatment of animal diseases or the presence of antibiotic residues in milk or the failure to observe withdrawal period (19), but an improvement was observed in the dairy farmers' awareness of antibiotics and AMR during this follow-up survey. In this survey, many of them were aware of the fact that antibiotics are used to treat bacterial infections, antibiotic residues can end up in milk, resistant bacteria can develop in the animal, and transfer to the humans. It was noticed that the knowledge score was higher in the farmers who took part in the first focus group discussion. It has been pointed out in a number of studies conducted in India, that the dairy farmers lack basic knowledge about antibiotics and often use antibiotics in the animals without the prescription or the involvement of any veterinarian (18, 31). It has further been suggested that there is an urgent need for periodic educational programs to instruct farmers on antibiotics, rational drug use, and resistance, as well as alternative measures including vaccination and preventive medicine (18, 32). In our study, just discussing these topics with dairy farmers raised their knowledge score, as evident by the fact that participation in the previous meeting had a significant effect, no matter the training received. Also, the farmers who were a part of intervention 2 (animal health) and 3 (animal health and simplified conventional AMR) had a higher knowledge score, probably because the discussions and interventions in them focused on preventive practices, management of common animal diseases like mastitis, foot, and mouth disease, zoonotic diseases as well as antibiotic use, AMR and this engrossed the farmers more with a better understanding. When designing training interventions, it is therefore important to make sure they are anchored in concepts the farmers understand and are interested in.

Regardless of the intervention, most dairy farmers believed that it is always best to call a veterinarian if the animal gets sick. Similar results are seen in another study conducted in Punjab (India), where the dairy farmers perceived veterinarians to be the most credible source as far as treatment of animals was concerned (33). However, previous results revealed that there is a shortage of veterinarians, and not all farmers have an easy access to veterinary and animal health related services, and this is the main reason, the farmers tend to treat the animals on their own, buying and using antibiotics without any prescription or seek the advice of informal prescribers (quacks) (19). Similar results were revealed in another study conducted in the Indian states Ludhiana, Guwahati, and Bangalore where an acute shortage of veterinarians led to irrational usage of antibiotics by the dairy farmers or the informal prescribers (quacks) (18). It is apparent from the findings obtained in this study that farmers think it is preferable to visit a veterinarian if the animals become sick, but many may not do so due to a lack of veterinarians in their area. Also, many of the farmers irrespective of the intervention approach stated that they do not follow withdrawal period when animals are on antibiotics and do not throw away the milk. Similar results were observed in other studies conducted in India where farmers are unaware of the concept of withdrawal period and do not discard the milk when the animal is on treatment (18, 34), and cite a number of reasons for not following the withdrawal period, where the major reason being the financial loss incurred if the milk of the treated animal was discarded (33).

Most of the veterinarians and paravets in the follow up survey also participated in the KIIs and received different interventions. Irrespective of the kind of intervention approach they were a part of, main thing that they remembered were the discussions about antibiotic use and AMR. The knowledge level of both the veterinarians and the paravets regarding antibiotics, AMR was found to be okay, but they did not really know how to explain the whole concept to the farmers. Some of them even felt that there is no need of explaining this to the farmers as they would not understand. Similar results were found in another study conducted in India to assess the knowledge, attitude, and practices of veterinarians relating to antimicrobial use, where most of the respondents had an average knowledge score regarding antibiotic use and AMR, however those veterinarians believed in educating farmers on good management practices for reducing antimicrobial use and had conducted training programs for the farmers to improve their knowledge on antibiotic usage (35). The main issue as per the veterinarians in combating AMR is the lack of awareness amongst the farmers, not completing the course of antibiotics and quacks giving antibiotics to the farmers for treating sick animals. Similar results were seen in the FGDs (19) and in many other studies conducted in India where the majority of the veterinarians attribute unauthorized practitioners (commonly called “quacks”) responsible for irrational use of antimicrobials and reported non-cooperation of the farmers in the completion of the antibiotic course prescribed by them (18, 35, 36).

## Conclusion

To conclude, regardless of any intervention, mere discussions on common animal diseases, preventive measures, antibiotic use seems to increase the knowledge level of the dairy farmers. The intervention techniques (2, 3), in which farmers were provided information on preventive measures for common diseases like mastitis etc. were more effective because mastitis is a significant problem in India and impacts them financially, making them more interested in the topic. The dairy farmers clearly believed in the advice given by the veterinarians when it comes to the animal health concerns but maybe because of the lack of enough veterinarians, farmers tend to treat the animals on their own or reach out to the quacks for help. To adequately address the issue, veterinary human resources must be strengthened in terms of both quantity and capability. The efficient transmission of information from veterinarians to farmers can lead to a considerable shift in farmers' attitudes toward antibiotic use and decreased reliance on quacks. The paravets are mostly involved in the treatment of mild infections with the fewest technicalities. In India, paravets are usually allowed to perform artificial insemination at farmers' homes, and being a local, they can add a lot of value in information dissemination. Both the veterinarians and paravets should be given frequent trainings on the topic and on how to make the farmers understand leading to a behavioral change. The need of the hour is for a “One Health” approach that facilitates behavioral change interventions in farmers and veterinarians, paravets by bringing all stakeholders together and promoting cautious antimicrobial usage and judicious antimicrobial stewardship.

## Data Availability Statement

The raw data supporting the conclusions of this article will be made available by the authors, without undue reservation.

## Ethics Statement

The study was approved by the Institutional Research Ethics Committee (IREC) at International Livestock Research Institute (ILRI), ILRI-IREC 2018-25. All the participants (farmers, veterinarians, paravets) were informed about the study and written consents were obtained from them prior to the filling of questionnaires in the follow up survey. The patients/participants provided their written informed consent to participate in this study.

## Author Contributions

JL: conceptualization, fund acquisition, investigation, methodology, management, and supervision. GS: investigation, methodology, and wrote the first draft. FM: investigation and methodology. RD: methodology and management. DG: conceptualization and fund acquisition. All authors revised the manuscript and have approved.

## Funding

This project was supported by the ICAR-ILRI Collaboration Funds and the CGIAR Research Program Agriculture for Nutrition and Health. This publication was also supported by the CGIAR Initiative-Protecting human health through a One Health approach and the contributors to the CGIAR Trust Fund (https://www.cgiar.org/funders/).

## Conflict of Interest

The authors declare that the research was conducted in the absence of any commercial or financial relationships that could be construed as a potential conflict of interest.

## Publisher's Note

All claims expressed in this article are solely those of the authors and do not necessarily represent those of their affiliated organizations, or those of the publisher, the editors and the reviewers. Any product that may be evaluated in this article, or claim that may be made by its manufacturer, is not guaranteed or endorsed by the publisher.
