# Supplementary material for: Comparing the Effectiveness of Different Approaches to Raise Awareness About Antimicrobial Resistance in Farmers and Veterinarians of India
Source: Front Public Health. 2022 Jun 16;10:837594. doi: 10.3389/fpubh.2022.837594 (PMC9244170; doi:10.3389/fpubh.2022.837594)
Supplement: Supplementary file 1 [file Presentation_1.PPTX]

## Slide 1
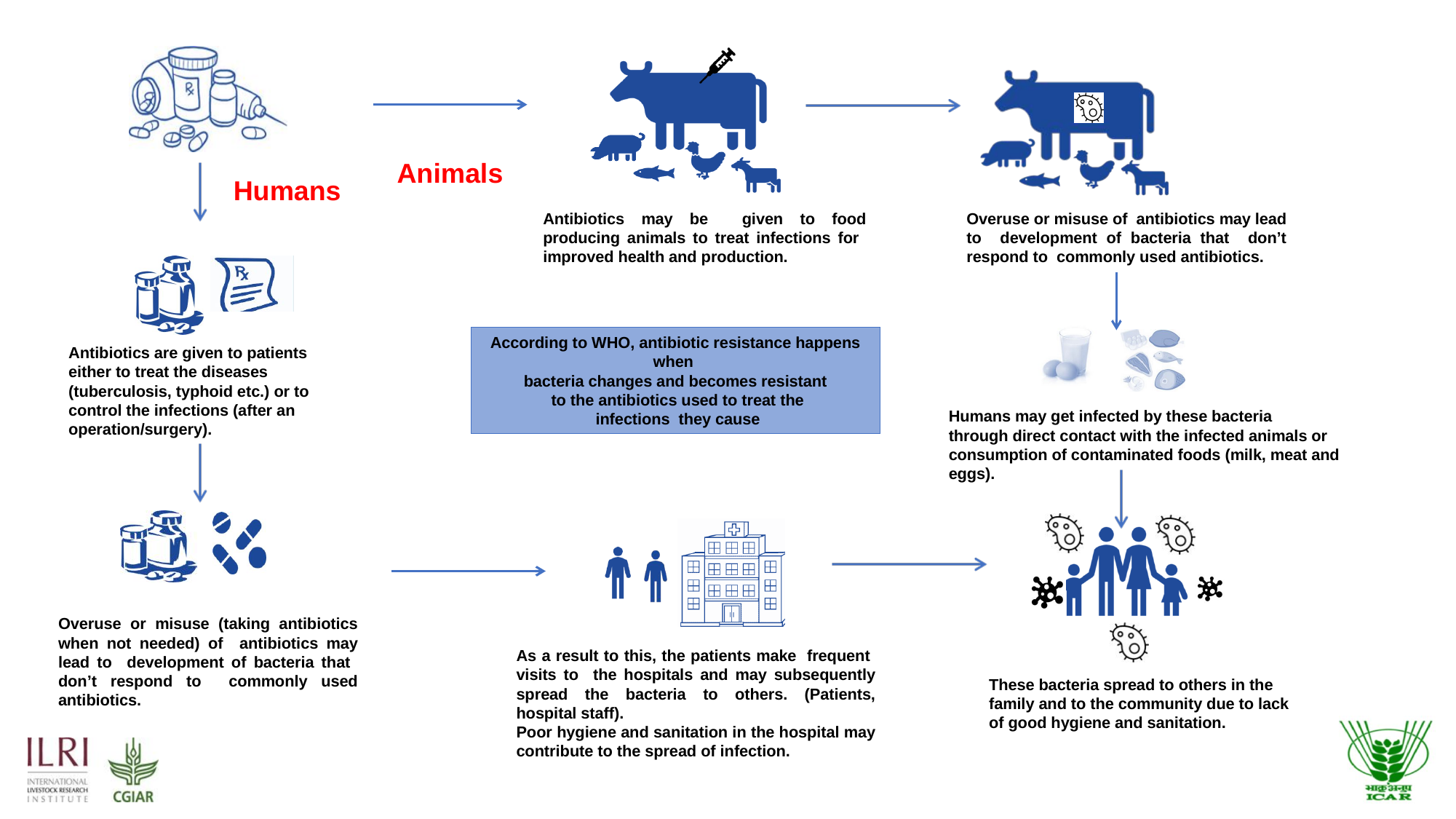

Animals
Humans
Antibiotics may be given to food producing animals to treat infections for improved health and production.
Overuse or misuse of antibiotics may lead to development of bacteria that don’t respond to commonly used antibiotics.
According to WHO, antibiotic resistance happens when
bacteria changes and becomes resistant
 to the antibiotics used to treat the
 infections they cause
Antibiotics are given to patients either to treat the diseases (tuberculosis, typhoid etc.) or to control the infections (after an operation/surgery).
Humans may get infected by these bacteria
through direct contact with the infected animals or consumption of contaminated foods (milk, meat and eggs).
Overuse or misuse (taking antibiotics when not needed) of antibiotics may lead to development of bacteria that don’t respond to commonly used antibiotics.
As a result to this, the patients make frequent visits to the hospitals and may subsequently spread the bacteria to others. (Patients, hospital staff).
Poor hygiene and sanitation in the hospital may contribute to the spread of infection.
These bacteria spread to others in the family and to the community due to lack of good hygiene and sanitation.

## Slide 2
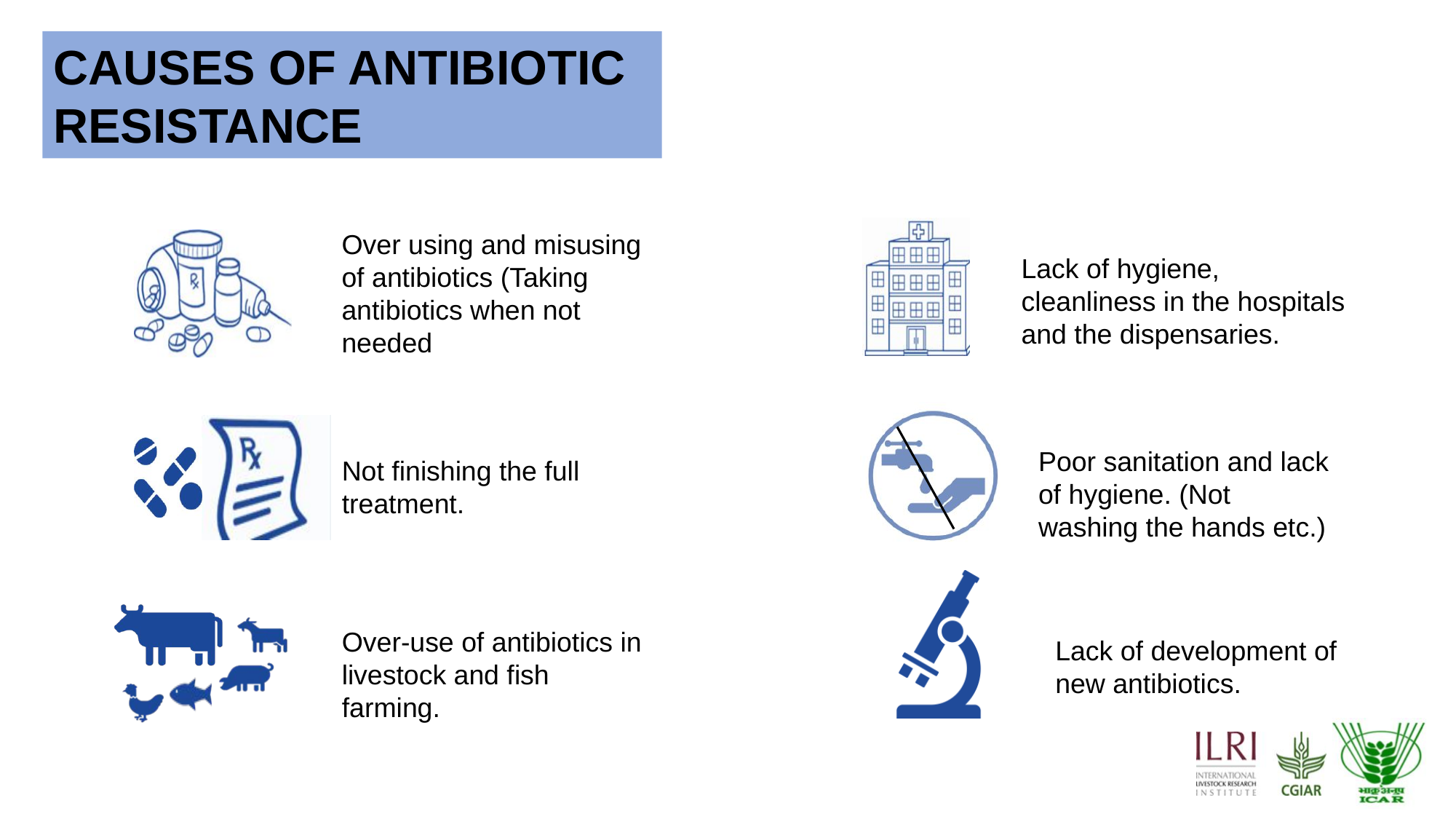

CAUSES OF ANTIBIOTIC RESISTANCE
Over using and misusing of antibiotics (Taking antibiotics when not needed
Lack of hygiene, cleanliness in the hospitals and the dispensaries.
Poor sanitation and lack of hygiene. (Not washing the hands etc.)
Not finishing the full treatment.
Over-use of antibiotics in livestock and fish farming.
Lack of development of new antibiotics.

## Slide 3
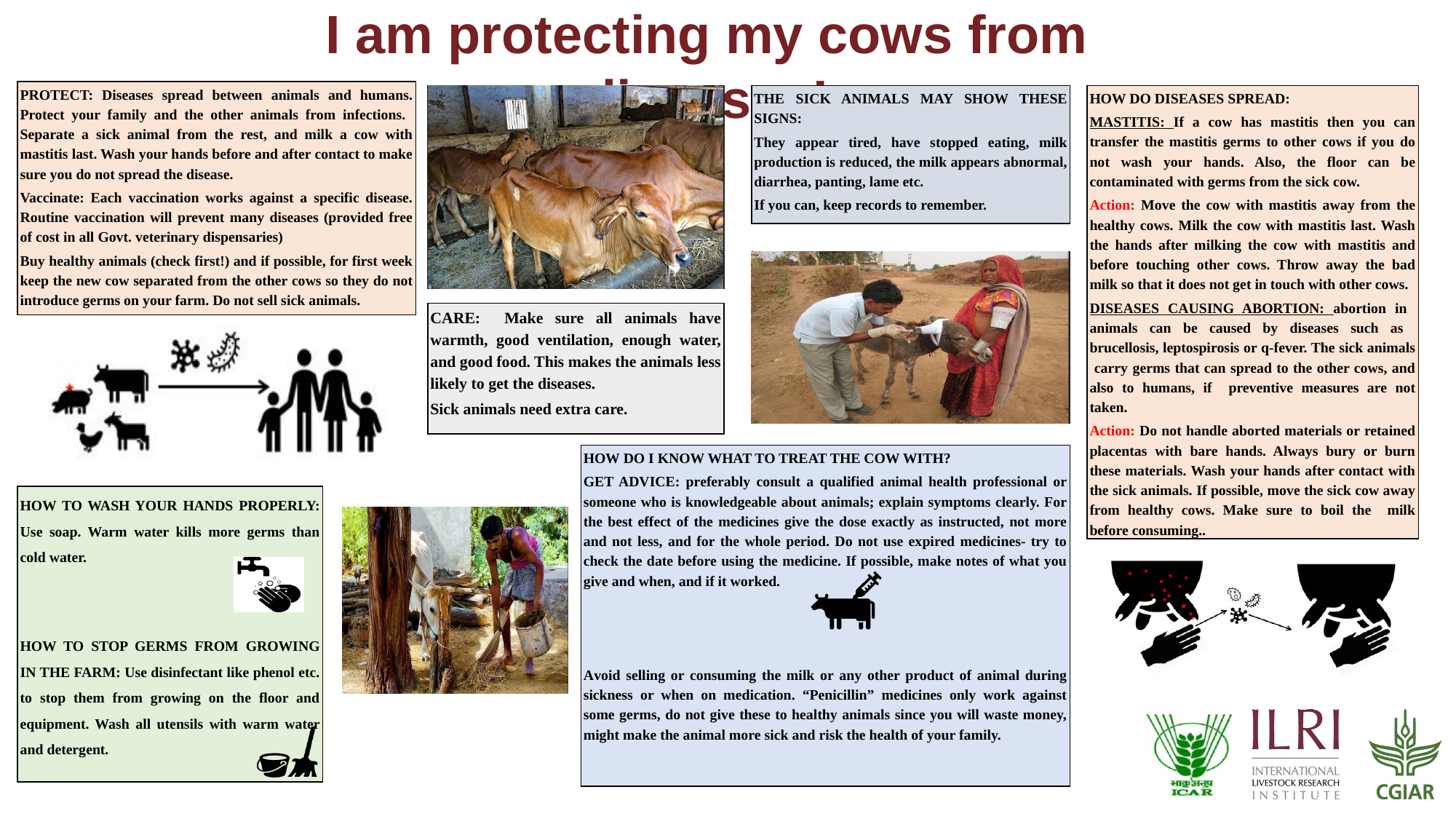

I am protecting my cows from diseases!
PROTECT: Diseases spread between animals and humans. Protect your family and the other animals from infections. Separate a sick animal from the rest, and milk a cow with mastitis last. Wash your hands before and after contact to make sure you do not spread the disease.
Vaccinate: Each vaccination works against a specific disease. Routine vaccination will prevent many diseases (provided free of cost in all Govt. veterinary dispensaries)
Buy healthy animals (check first!) and if possible, for first week keep the new cow separated from the other cows so they do not introduce germs on your farm. Do not sell sick animals.
THE SICK ANIMALS MAY SHOW THESE SIGNS:
They appear tired, have stopped eating, milk production is reduced, the milk appears abnormal, diarrhea, panting, lame etc.
If you can, keep records to remember.
HOW DO DISEASES SPREAD:
MASTITIS: If a cow has mastitis then you can transfer the mastitis germs to other cows if you do not wash your hands. Also, the floor can be contaminated with germs from the sick cow.
Action: Move the cow with mastitis away from the healthy cows. Milk the cow with mastitis last. Wash the hands after milking the cow with mastitis and before touching other cows. Throw away the bad milk so that it does not get in touch with other cows.
DISEASES CAUSING ABORTION: abortion in animals can be caused by diseases such as brucellosis, leptospirosis or q-fever. The sick animals carry germs that can spread to the other cows, and also to humans, if preventive measures are not taken.
Action: Do not handle aborted materials or retained placentas with bare hands. Always bury or burn these materials. Wash your hands after contact with the sick animals. If possible, move the sick cow away from healthy cows. Make sure to boil the milk before consuming..
CARE: Make sure all animals have warmth, good ventilation, enough water, and good food. This makes the animals less likely to get the diseases.
Sick animals need extra care.
HOW DO I KNOW WHAT TO TREAT THE COW WITH?
GET ADVICE: preferably consult a qualified animal health professional or someone who is knowledgeable about animals; explain symptoms clearly. For the best effect of the medicines give the dose exactly as instructed, not more and not less, and for the whole period. Do not use expired medicines- try to check the date before using the medicine. If possible, make notes of what you give and when, and if it worked.
Avoid selling or consuming the milk or any other product of animal during sickness or when on medication. “Penicillin” medicines only work against some germs, do not give these to healthy animals since you will waste money, might make the animal more sick and risk the health of your family.
HOW TO WASH YOUR HANDS PROPERLY: Use soap. Warm water kills more germs than cold water.
HOW TO STOP GERMS FROM GROWING IN THE FARM: Use disinfectant like phenol etc. to stop them from growing on the floor and equipment. Wash all utensils with warm water and detergent.

## Slide 4
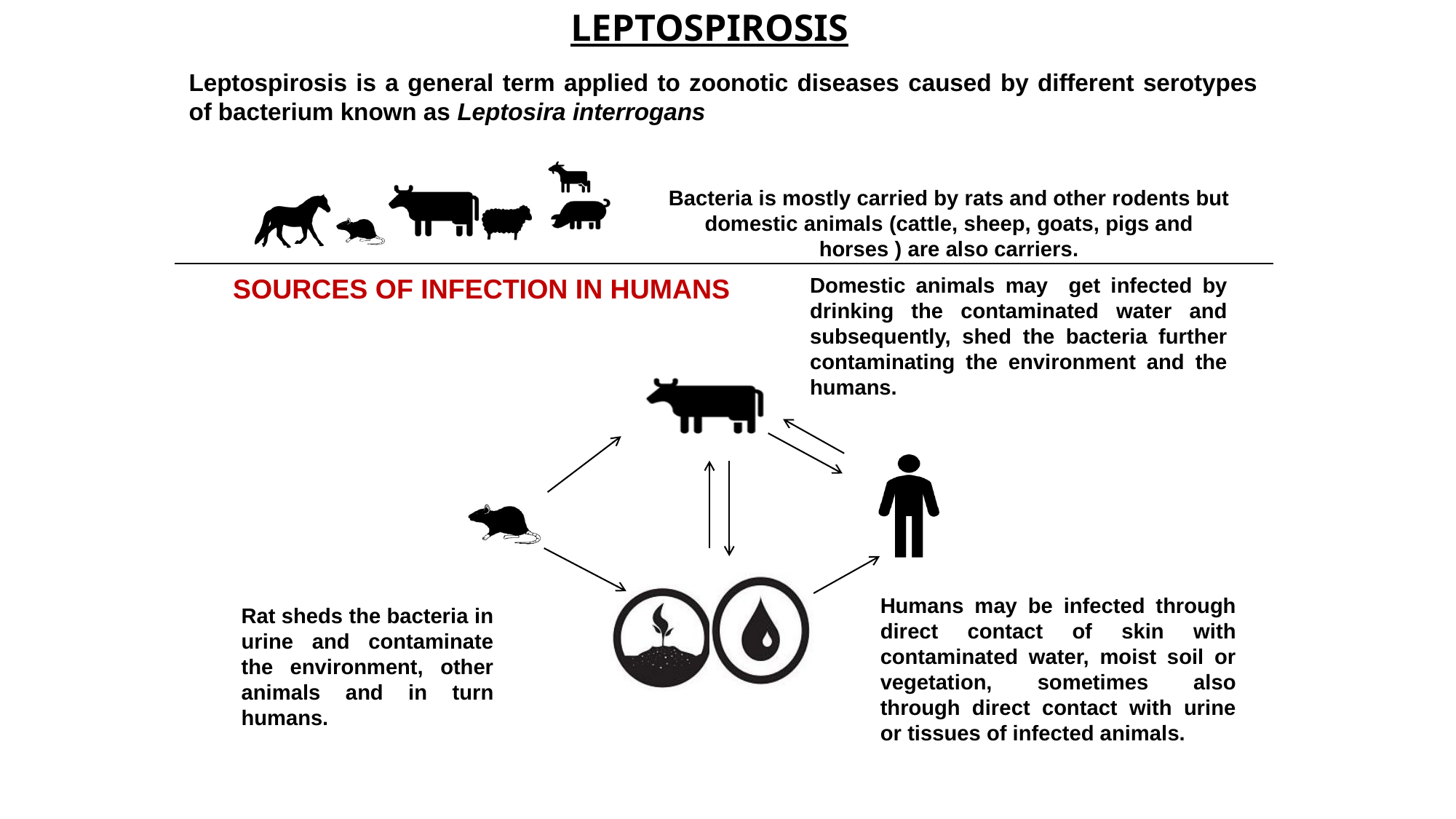

LEPTOSPIROSIS
Leptospirosis is a general term applied to zoonotic diseases caused by different serotypes of bacterium known as Leptosira interrogans
Bacteria is mostly carried by rats and other rodents but domestic animals (cattle, sheep, goats, pigs and horses ) are also carriers.
SOURCES OF INFECTION IN HUMANS
Domestic animals may get infected by drinking the contaminated water and subsequently, shed the bacteria further contaminating the environment and the humans.
Humans may be infected through direct contact of skin with contaminated water, moist soil or vegetation, sometimes also through direct contact with urine or tissues of infected animals.
Rat sheds the bacteria in urine and contaminate the environment, other animals and in turn humans.

## Slide 5
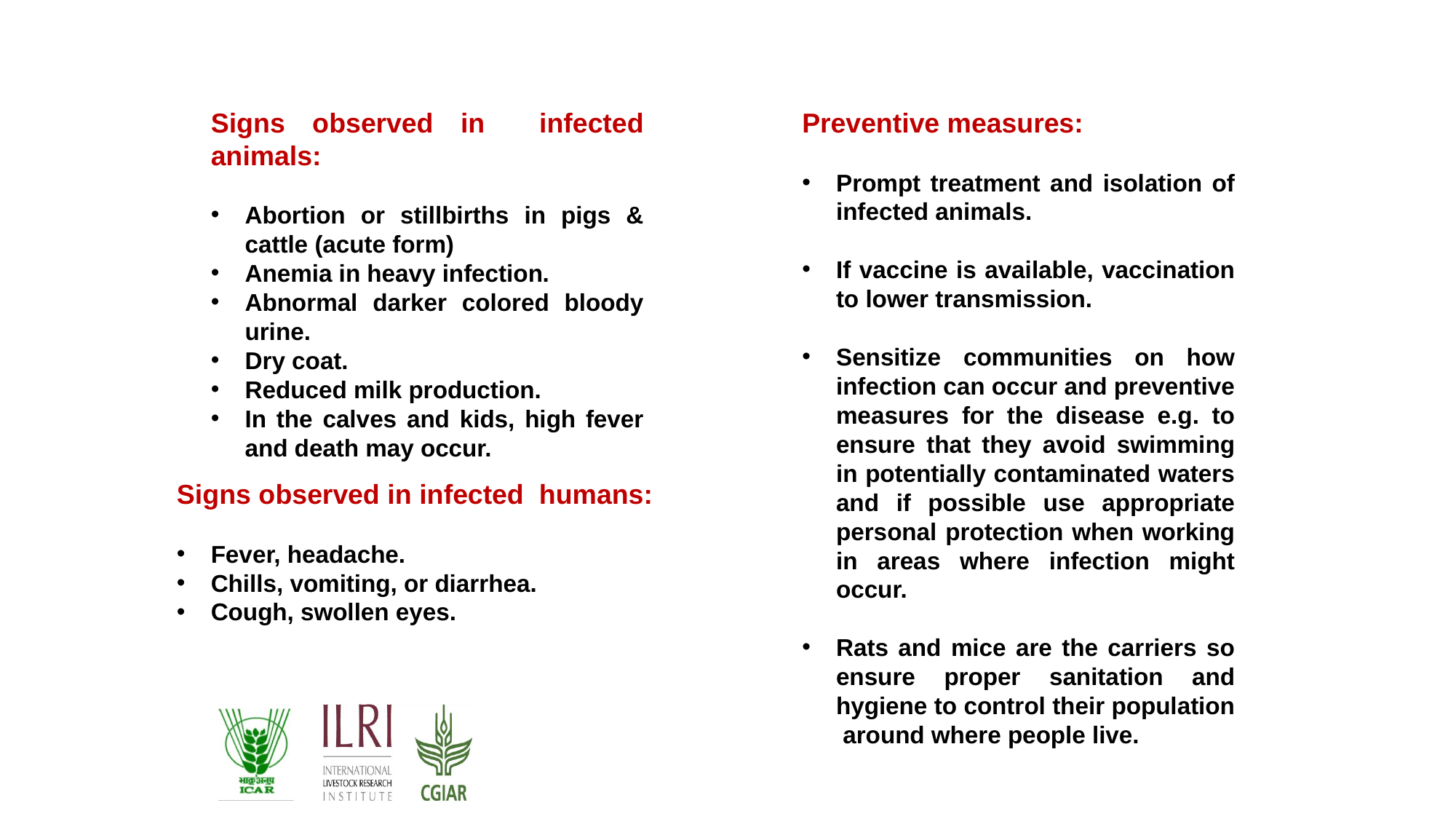

Signs observed in infected animals:
Abortion or stillbirths in pigs & cattle (acute form)
Anemia in heavy infection.
Abnormal darker colored bloody urine.
Dry coat.
Reduced milk production.
In the calves and kids, high fever and death may occur.
Preventive measures:
Prompt treatment and isolation of infected animals.
If vaccine is available, vaccination to lower transmission.
Sensitize communities on how infection can occur and preventive measures for the disease e.g. to ensure that they avoid swimming in potentially contaminated waters and if possible use appropriate personal protection when working in areas where infection might occur.
Rats and mice are the carriers so ensure proper sanitation and hygiene to control their population around where people live.
Signs observed in infected humans:
Fever, headache.
Chills, vomiting, or diarrhea.
Cough, swollen eyes.

## Slide 6
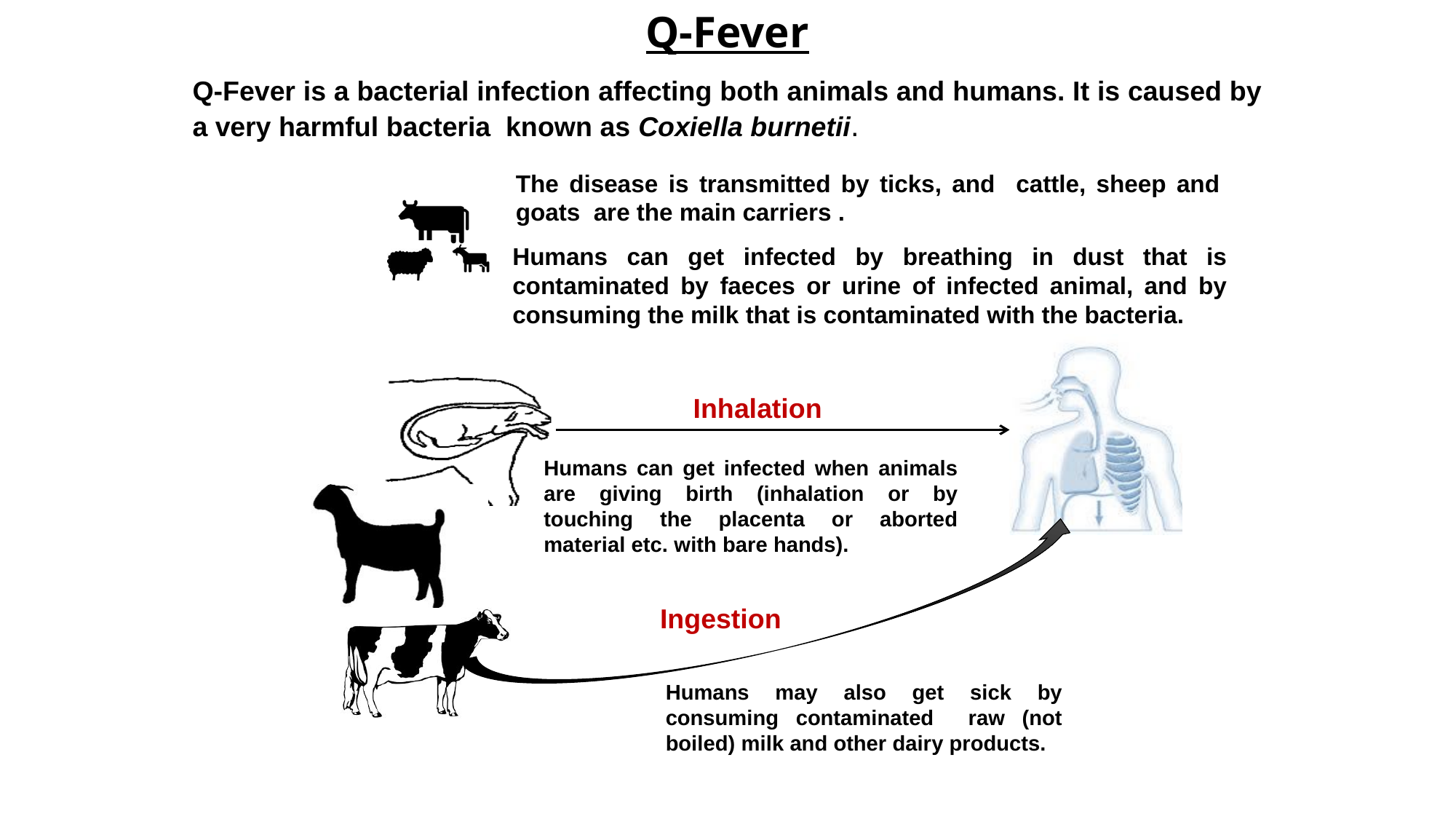

Q-Fever
Q-Fever is a bacterial infection affecting both animals and humans. It is caused by a very harmful bacteria known as Coxiella burnetii.
The disease is transmitted by ticks, and cattle, sheep and goats are the main carriers .
Humans can get infected by breathing in dust that is contaminated by faeces or urine of infected animal, and by consuming the milk that is contaminated with the bacteria.
Inhalation
Humans can get infected when animals are giving birth (inhalation or by touching the placenta or aborted material etc. with bare hands).
Ingestion
Humans may also get sick by consuming contaminated raw (not boiled) milk and other dairy products.

## Slide 7
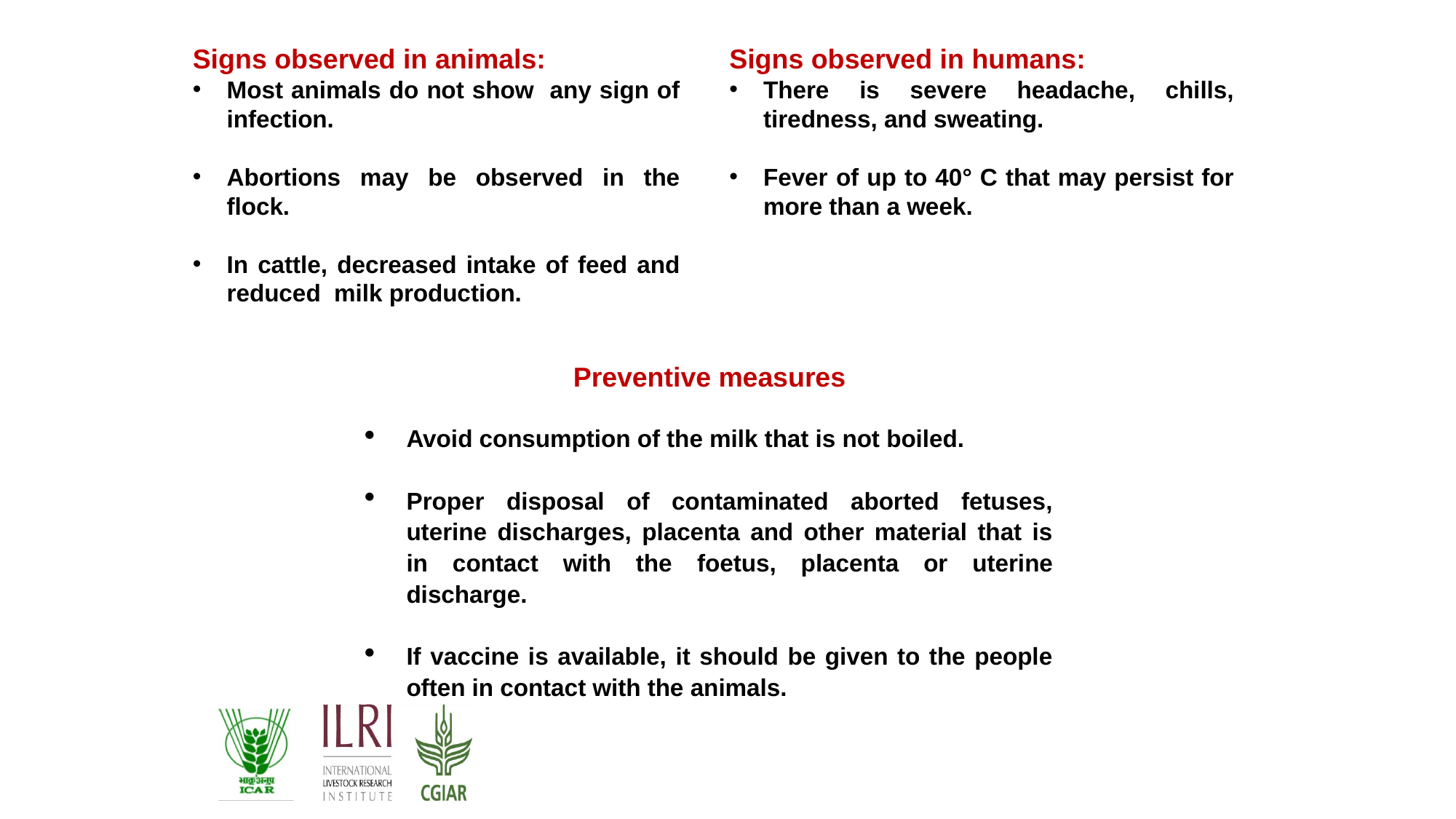

Signs observed in humans:
There is severe headache, chills, tiredness, and sweating.
Fever of up to 40° C that may persist for more than a week.
Signs observed in animals:
Most animals do not show any sign of infection.
Abortions may be observed in the flock.
In cattle, decreased intake of feed and reduced milk production.
Preventive measures
Avoid consumption of the milk that is not boiled.
Proper disposal of contaminated aborted fetuses, uterine discharges, placenta and other material that is in contact with the foetus, placenta or uterine discharge.
If vaccine is available, it should be given to the people often in contact with the animals.

## Slide 8
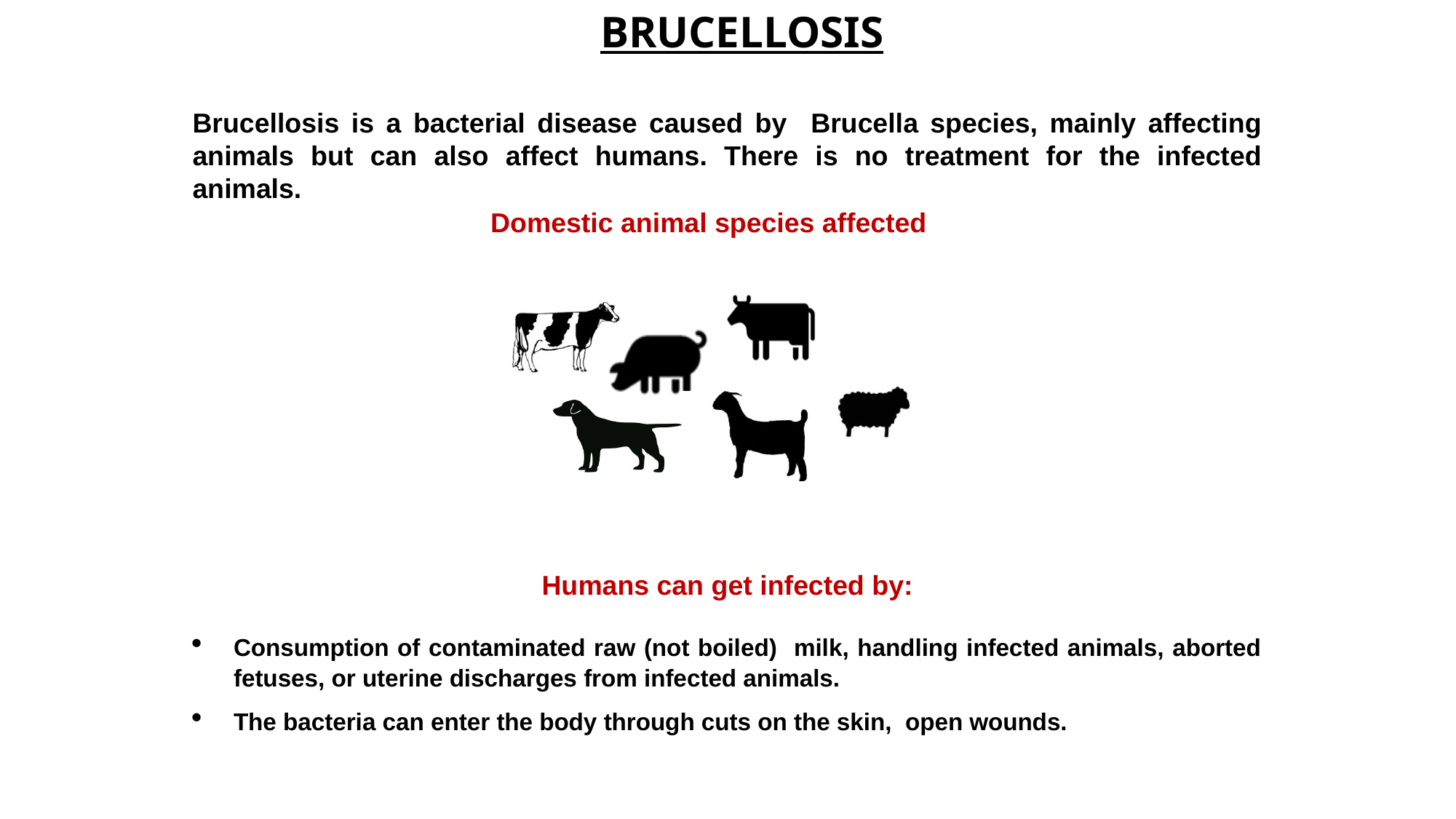

BRUCELLOSIS
Brucellosis is a bacterial disease caused by Brucella species, mainly affecting animals but can also affect humans. There is no treatment for the infected animals.
Domestic animal species affected
Humans can get infected by:
Consumption of contaminated raw (not boiled) milk, handling infected animals, aborted fetuses, or uterine discharges from infected animals.
The bacteria can enter the body through cuts on the skin, open wounds.

## Slide 9
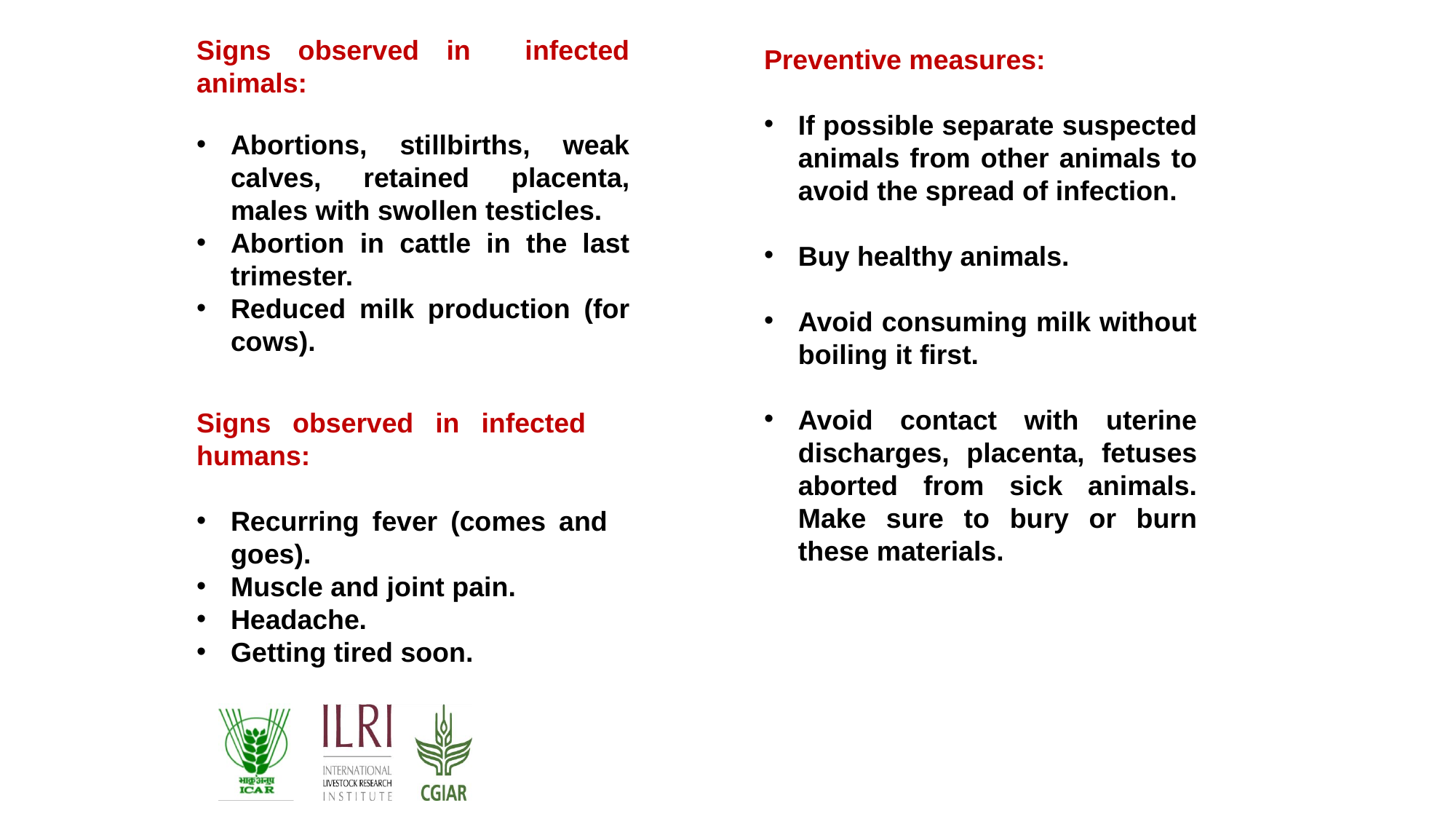

Signs observed in infected animals:
Abortions, stillbirths, weak calves, retained placenta, males with swollen testicles.
Abortion in cattle in the last trimester.
Reduced milk production (for cows).
Preventive measures:
If possible separate suspected animals from other animals to avoid the spread of infection.
Buy healthy animals.
Avoid consuming milk without boiling it first.
Avoid contact with uterine discharges, placenta, fetuses aborted from sick animals. Make sure to bury or burn these materials.
Signs observed in infected humans:
Recurring fever (comes and goes).
Muscle and joint pain.
Headache.
Getting tired soon.

## Slide 10
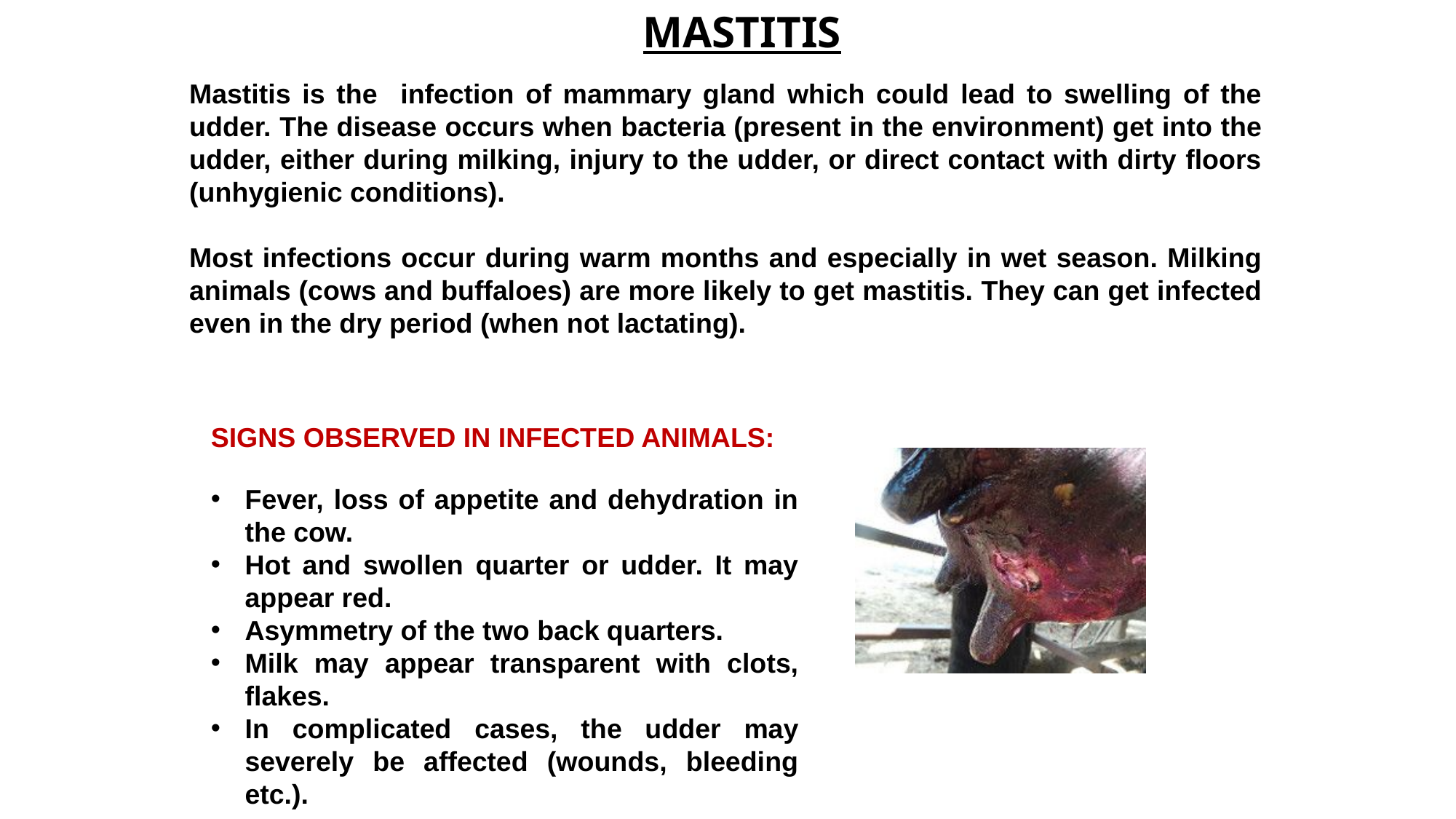

MASTITIS
Mastitis is the infection of mammary gland which could lead to swelling of the udder. The disease occurs when bacteria (present in the environment) get into the udder, either during milking, injury to the udder, or direct contact with dirty floors (unhygienic conditions).
Most infections occur during warm months and especially in wet season. Milking animals (cows and buffaloes) are more likely to get mastitis. They can get infected even in the dry period (when not lactating).
SIGNS OBSERVED IN INFECTED ANIMALS:
Fever, loss of appetite and dehydration in the cow.
Hot and swollen quarter or udder. It may appear red.
Asymmetry of the two back quarters.
Milk may appear transparent with clots, flakes.
In complicated cases, the udder may severely be affected (wounds, bleeding etc.).

## Slide 11
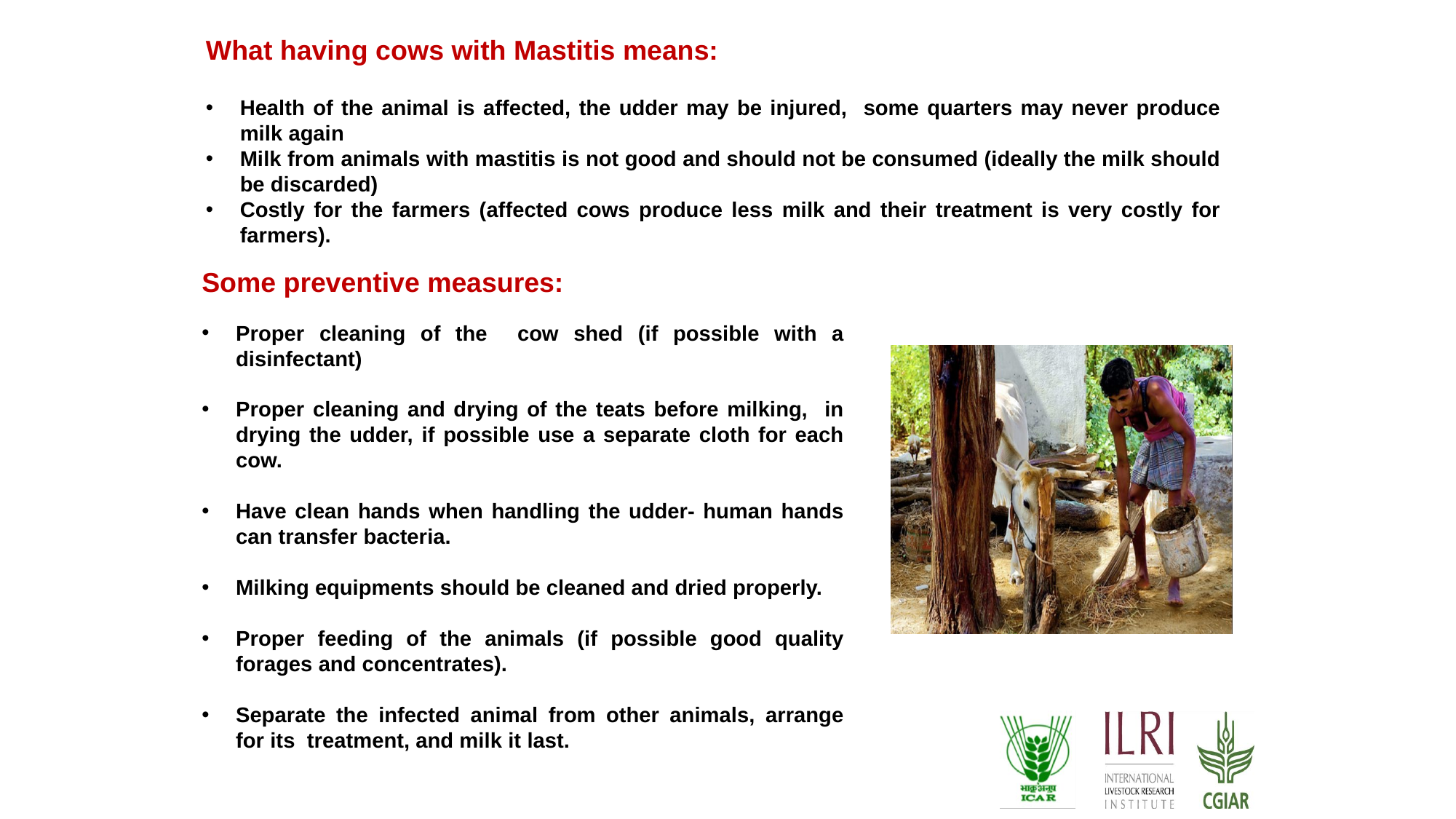

What having cows with Mastitis means:
Health of the animal is affected, the udder may be injured, some quarters may never produce milk again
Milk from animals with mastitis is not good and should not be consumed (ideally the milk should be discarded)
Costly for the farmers (affected cows produce less milk and their treatment is very costly for farmers).
Some preventive measures:
Proper cleaning of the cow shed (if possible with a disinfectant)
Proper cleaning and drying of the teats before milking, in drying the udder, if possible use a separate cloth for each cow.
Have clean hands when handling the udder- human hands can transfer bacteria.
Milking equipments should be cleaned and dried properly.
Proper feeding of the animals (if possible good quality forages and concentrates).
Separate the infected animal from other animals, arrange for its treatment, and milk it last.
