# Supplementary material for: Comparing the Effectiveness of Different Approaches to Raise Awareness About Antimicrobial Resistance in Farmers and Veterinarians of India
Source: Front Public Health. 2022 Jun 16;10:837594. doi: 10.3389/fpubh.2022.837594 (PMC9244170; doi:10.3389/fpubh.2022.837594)
Supplement: Supplementary file 2 [file Data_Sheet_1.pdf]

FGD Guide for the farmers

Greetings! Thank you for your willingness to participate in the discussion. I am________________, from International livestock research institute (ILRI). I have planned this discussion to know your perspective about animal health and antibiotic use. I am just going to ask you a few questions related to the study. You may not benefit directly from the study but the information you provide will help us frame further interventions.

1.)	**How important is livestock rearing for you?**<br>***Probes:***<br>• For what purposes do they use animals?<br>• What are the common breeds?<br>• What are the common challenges do you sface in rearing the animals?
2.)	**Which are the most common animal health issues in this community (cattle, buffaloes, sheep / goats, poultry) ---*listing***<br>***Probes:***<br>• What causes disease?<br>• How frequent is the occurrence of the disease?
3.)	**What do you do when you observe a sick animal in your farm? –*describe***<br>***Probes:***<br>• Treat on my own, get advice from neighbours; call an animal health professional, do nothing etc.? *(can score each option)*<br>• Probe for animal health consultation--who is consulted (quack / vet), when consulted? Nature of assistance given (phone call advise, visits, etc.)<br>• Selling the animals (where sold, who buys (farmers, traders), sell price (lower, usual etc.). --why would one want to sell?<br>• Other products used (traditional medicine etc.)
4.)	**When your animal gets sick, do you give any medicines to them?**<br>***Probes:***<br>• What kind of medicines?<br>• Their source-----neighbors, OTC?<br>• How do they determine dosage?
4.)	**Is there any risk of using medicines in food animals? If yes, which one?**<br>***Probes:***

	• What do you do with milk from cows / buffaloes produced by cows that are sick? What about from sick animals those are on treatment?<br>• Probe to see if they are aware of antibiotic residues in milk and their effects when consumed.<br>• Probe to see if they are aware of drug withdrawal periods (do they observe, for what products, what are their perceptions)
5.)	**Are you aware of any diseases that people can get from sick animals? If yes, which ones? Can you name some?**<br><br>***Probes:***<br><br>• How can the infection occur from animals to humans? (The possible routes of transmission)<br>• What are the preventive measures you should take while handling a sick animal?<br>• Abortion in animals, is it common in this village? Are you aware any disease that would cause abortions in domestic animals? How do you handle the aborted foetus?
6.)	**Do you vaccinate your animals?**<br><br>**Probes:**<br><br>• Do you know what vaccination does?<br>• Who comes for vaccinating your animals?<br>• Do you pay for vaccination?
7.)	**Do you keep any records? If yes, which one(s)?**<br><br>***Probes:***<br><br>• Probe for health records (e.g. medication given to sick animals, when given, by who etc.).<br>• Production records (e.g. milk production etc.).
