# Supplementary material for: Comparing the Effectiveness of Different Approaches to Raise Awareness About Antimicrobial Resistance in Farmers and Veterinarians of India
Source: Front Public Health. 2022 Jun 16;10:837594. doi: 10.3389/fpubh.2022.837594 (PMC9244170; doi:10.3389/fpubh.2022.837594)
Supplement: Supplementary file 3 [file Data_Sheet_2.pdf]

KII Guide for the Veterinarians

Greetings! Thank you for your willingness to participate in the interview. I am_________________, from International livestock research institute (ILRI). I have planned this discussion to know your perspective about animal health and antibiotic use. I am just going to ask you a few questions related to the study. You may not benefit directly from the study but the information you provide will help us frame further interventions.

1.)	**Since how long have you been in the veterinary practice? What are your key responsibilities?**<br><br>***Probes:***<br><br>• No. of animal cases attended in a week<br>• Do farmers call you directly or are there cases of referrals?<br>• Kind of diseases encountered in the livestock animals (common diseases)
2.)	**Antibiotic prescriptions: How often do you prescribe antibiotics?**<br><br>***Probes:***<br><br>• Which antibiotic do you prescribe most of the time? Why? Are there some you don't prescribe at all? Why?<br>• Where do you source the drugs from (Government supply? buy from a nearby pharmaceutical shop? Or a particular pharmaceutical shop? etc.<br>• Are there times you get the drug on credit from the pharmacy, then you pay after using it at the farm?
3.)	**How does the antibiotic use in the animals affect human health? Do you think there is any link between the two?**<br><br>***Probes:***<br><br>• Are you aware about anti-microbial resistance? (Antibiotic residue levels, likely causes of AMR in India, effects etc.)<br>• How did you come to know about AMR?<br>• To what extent are farmers, quacks, veterinarians, pharmacists, pharmaceutical companies, policies etc. to blame for increasing levels of AMR?<br>• When you prescribe (or sell OTC for pharmacists) antibiotics, do you usually discuss with farmers about withdrawal periods, in your own view, do they follow?<br>• Are you aware of any common antibiotic that has become ineffective as a result of bacteria becoming resistant to it?
4.)	**Can you elaborate a bit upon the preventive measures that can be used to reduce the occurrence of diseases and use of antibiotics?**<br><br>***Probes:***<br>• Vaccination<br>• Improving feed quality<br>• Maintaining hygiene<br>• Sending samples to the laboratory before prescribing.

5.)	**How do you tell the farmers about the importance of preventive measures while dealing with the sick animals?**<br>***Probes:***<br>• Are there any trainings or workshops arranged?<br>• IEC activity etc.
6.)	**What are the common zoonotic diseases observed in the area?**<br><br>***Probes:***<br>• How do you diagnose?<br>• What preventive measures do you take while handling the animal with a zoonotic infection?<br>• Do you tell the farmer about the proper method of disposing the aborted material and other preventive measure to be taken?
7.)	**Is routine vaccination of livestock animals carried out in your area?**<br><br>***Probes:***<br>• Who is responsible for the vaccination?<br>• From where do you get the vaccines?<br>• Are the farmers made aware about the importance of vaccination?<br>• Are the farmers charged for vaccinating their animals?
