# Supplementary material for: Comparing the Effectiveness of Different Approaches to Raise Awareness About Antimicrobial Resistance in Farmers and Veterinarians of India
Source: Front Public Health. 2022 Jun 16;10:837594. doi: 10.3389/fpubh.2022.837594 (PMC9244170; doi:10.3389/fpubh.2022.837594)
Supplement: Supplementary file 4 [file Data_Sheet_3.pdf]

Survey No. / Questionnaire No.____________

**MANAGING THE SPREAD OF INFECTIOUS DISEASES AND AMR THROUGH AWARENESS CREATION AND COMMUNITY SENSITIZATION**

This questionnaire will take approximately 20 minutes to answer. Please, be assured that any Information you provide will be anonymous and no personal information collected will appear In any documents or reports based on this survey.

**Part-1**

**Profiling**

| | |
|---|---|
| **State** | |
| **District** | |
| **Name of the village** | |
| **Type of farmer** | • Smallholder dairy farm (<10 milking cows/buffaloes) **(1)**<br>• Commercial dairy farm (>10 milking cows/buffaloes) **(0)** |
| **How many cows/buffaloes do you have** | • In total______<br>• Milking______ |
| **How many cows have been sick the last month** | |
| **Which medicines have you used for your animals the last months?** | |

**Part-2**

**Socio-demographic characteristics**

| | |
|---|---|
| **Gender** | • Male **(1)**<br>• Female **(0)** |
| **Which is the highest level of education you have finalized?** | • Basic education till 10th **(1)**<br>• Senior secondary **(2)**<br>• Professional education **(3)**<br>• Diploma holder **(4)**<br>• None of the above **(0)** |
| **How old are you?** | ______ years |

**Did you participate in any of our previous focus group meetings? (Yes=1; No=0); If yes, briefly tell us what you can remember from that training?**

| |
|---|
| |

**Referent beliefs**

| | |
|---|---|
| **3.1) Last time you had a sick animal, what did you do as a first alternative?** | • Call a paravet, veterinary Doctor or veterinary field assistant **(1)**<br>• Take the help of neighbors **(2)**<br>• Got advice from the local pharmacist **(3)**<br>• Treat on my own (with an old medicine or buy medicine without an advice) **(4)**<br>• Others **(5)** |
| **3.2) If you called a paravet, veterinary Doctor or veterinary field assistant, what kind of assistance was given?** | • Paid visit to the farm **(1)**<br>• Phone call advise **(2)**<br>• Other **(3), please describe_________** |
| **3.3) What was the cost for the help you got?** | • Cost ____ INR |

**Part-4**

| | |
|---|---|
| **4.1) What are antibiotics?** | • Drugs that treat viral infection **(Correct (1) or incorrect (0)?)**<br>• Drugs that treat bacterial infection **(Correct (1) or incorrect (0)?)**<br>• Drugs that treat any infection **(Correct (1) or incorrect (0)?)**<br>• Drugs that make animals grow better **(Correct (1) or incorrect (0)?)**<br>• Drugs that stop animals from getting sick **(Correct (1) or incorrect (0)?)** |
| **4.2) Why do you think antimicrobials (drugs) are given to the animals?** | • To treat diseases: **Not common (0), Common (1), Very common (2)**<br>• To increase productivity: **Not common (0), Common (1), Very common (2)**<br>• To stop diseases from happening: **Not common (0), Common (1), Very common (2)** |
| **4.3) Do you think these things can happen when you give antibiotics to the farm animals?** | • Some antibiotic residues can get into the milk: **(not possible (0), unlikely (1), likely (2) very likely (3))**<br>• The germs infecting the animal can become resistant: **(not possible (0), unlikely (1), likely (2) very likely (3))**<br>• Resistant bacteria can develop in the animal, and transfer to the human: **(not possible (0), unlikely (1), likely (2) very likely (3))** |

**Part-5**

**Knowledge (Zoonotic diseases)**

| | |
|---|---|
| **5.1) Are you aware of any diseases that people can get from sick animals?** | • Yes **(1)**<br>• No **(0) (If this, skip 5.2)** |
| **5.2) Can you mention three diseases that people can get from animals?** | |
| **5.3) What is the mode of transmission of infection from animals to humans?** | • Contact: **Yes(1) No (0)**<br>• Inhalation: **Yes(1) No (0)**<br>• Ingestion: **Yes(1) No (0)**<br>• Contact with animal products: **Yes(1) No (0)** |
| **5.4) What are the preventive and control measures you should take while handling sick animal?** | • Separate the sick animal from healthy animals: **Yes(1) No (0)**<br>• Wear gloves (cover hands) while handling the sick animal: **Yes(1) No (0)**<br>• Wash hands with soap after touching the sick animal: **Yes(1) No (0)**<br>• Discard everything coming from the sick cow so that it is not in contact: **Yes(1) No (0)** |

**5.5) How worried are you about getting an infectious disease from your livestock?**

1. Not at all **(0)**
2. Very little **(1)**
3. A little bit **(2)**
4. Much **(3)**
5. very much **(4)**

**Part- 6**

**Perceived risk**

**How would you perceive the likelihood….**

| | |
|---|---|
| **6.1) That you can get infected with an antibiotic resistant bacteria if I go to a hospital:** | • Not possible **(0)**<br>• Very small **(1)** |

| | |
|---|---|
| | • Small **(2)**<br>• Big **(3)**<br>• Very big **(4)**<br>• Don't Know **(5)** |
| **6.2) That you can get infected with drug resistant bacteria through my livestock is:** | • Not possible **(0)**<br>• Very small **(1)**<br>• Small **(2)**<br>• Big **(3)**<br>• Very big **(4)**<br>• Don't Know **(5)** |
| **6.3) The chance I can get infected through diseased/sick animals:** | • Very small **(0)**<br>• Small **(1)**<br>• Big **(2)**<br>• Very big **(3)**<br>• No chance **(4)**<br>• Don't Know **(5)** |
| **6.4) How big is the problem of resistant bacteria** | • There is no problem **(0)**<br>• Very small **(1)**<br>• Small **(2)**<br>• Big **(3)**<br>• Very big **(4)**<br>• Don't Know **(5)** |

**Part- 7**

**Attitude (antimicrobial regulations)**

***I will list you two scenarios of talking to two hypothetical neighbours of your, and I would be grateful if you would tell me which one you agree the most with***

**7.1)**

**Neighbour 1 says:** It is good that antimicrobials for my animals are available without any prescription, so that I can treat them on my own.

**Neighbour 2 says:** I think it is best to always call a veterinarian so that the animal really gets the right treatment

**Who do you most agree with?**

- Strongly with neighbor 1 **(0)**
- A little bit more with neighbor 1 **(1)**
- Equally with both **(2)**

- A little bit with neighbor 2 **(3)**
- Strongly with neighbor 2 **(4)**

---

**7.2)**

**Neighbour 1 says:** If I don't give my animals antibiotics regularly, they will get sick and I will lose money.

**Neighbour 2 says:** We should stop giving so many antibiotics. I am worried that it will cause even more problems in the future if we do.

**Who do you most agree with?**

- Strongly with neighbor 1 **(0)**
- A little bit more with neighbor 1 **(1)**
- Equally with both **(2)**
- A little bit with neighbor 2 **(3)**
- Strongly with neighbor 2 **(4)**

---

**7.3)**

**Neighbour 1 says:** When the cow gets medicine, I always throw away the milk. There might be some of it in the milk.

**Neighbour 2 says:** I can't throw away the milk when I treat the animals; I have to sell it for money. I don't think it is so bad to drink it anyway.

**Who do you most agree with?**

- Strongly with neighbor 1 **(1)**
- A little bit more with neighbor 1 **(2)**
- Equally with both **(3)**
- A little bit with neighbor 2 **(4)**
- Strongly with neighbor 2 **(5)**

---

**7.4)**

**Neighbour 1 says:** There is no point contacting the veterinarian or the field assistant, I know more than them and it is cheaper to buy the medicine myself.

**Neighbour 2 says:** The veterinarian and the field assistant always give me the best advice.

**Who do you most agree with?**

- Strongly with neighbor 1 **(0)**
- A little bit more with neighbor 1 **(1)**
- Equally with both **(2)**
- A little bit with neighbor 2 **(3)**
- Strongly with neighbor 2 **(4)**

---
