# Supplementary material for: Comparing the Effectiveness of Different Approaches to Raise Awareness About Antimicrobial Resistance in Farmers and Veterinarians of India
Source: Front Public Health. 2022 Jun 16;10:837594. doi: 10.3389/fpubh.2022.837594 (PMC9244170; doi:10.3389/fpubh.2022.837594)
Supplement: Supplementary file 5 [file Data_Sheet_4.pdf]

Survey No. / Questionnaire No.____________

MANAGING THE SPREAD OF INFECTIOUS DISEASES AND AMR THROUGH AWARENESS CREATION AND COMMUNITY SENSITIZATION

This questionnaire will take approximately 20 minutes to answer. Please, be assured that any Information you provide will be anonymous and no personal information collected will appear In any documents or reports based on this survey.

Part-1

Profiling

**State**	
**District**	
**Name of the village**	
**Type of Practitioner**	• **Veterinary Doctor (1)**<br>• **Veterinary Pharmacist (2)**<br>• **Veterinary livestock assistant (3)**<br>• **Others (4)**

Part-2

Socio-demographic characteristics

**Gender**	• **Male (1)**<br>• **Female (0)**
**Education status**	• **Diploma Holder (1)**<br>• **Graduate (2)**<br>• **Post-graduate (3)**<br>• **Others (4) Please Describe_________**
**Age**	**________________years**

Did you participate in any of our previous meetings/interviews (in which extension material was given)? [Yes=1; No=0]; If yes, briefly tell us what you can remember from that conversation?

1.) Please explain to us how you would explain to a farmer what an antibiotic is?

2.) Please explain to us what do you understand by antibiotic resistance?

3.) Please explain to us how you would explain the concept of antibiotic resistance to a farmer?

4.) I will list you two scenarios of talking to two hypothetical colleagues of your, and I would be grateful if you would tell me which one you agree the most with

4.1)

Colleague 1 says: It is important to give the farmer antibiotics when he has a sick animal, otherwise he will not be happy and not call me again

Colleague 2 says: I think we should not give farmers so much antibiotics, only if we are really sure it is a bacteria causing the disease

How do you most agree with?

- Strongly with colleague 1 **(0)**

- A little bit more with colleague 1 **(1)**
- Equally with both **(2)**
- A little bit with colleague 2 **(3)**
- Strongly with colleague 2 **(4)**

4.2)

Colleague 1 says: I always tell the farmers that he must throw away the milk during medicine treatment and 2 days after, otherwise the milk can be harmful

Colleague 2 says: Farmers cannot afford to throw away milk during treatment, so I don't even tell them to do it.

How do you most agree with?

- Strongly with colleague 1 **(0)**
- A little bit more with colleague 1 **(1)**
- Equally with both **(2)**
- A little bit with colleague 2 **(3)**
- Strongly with colleague 2 **(4)**

5.)

*****Table for veterinarians and Para-vets***

5.1) How many cases have you attended to in the last one month? (…………………)

**Description of the two last reported cases of diseased/sick animals**	**Was it the farmer who called you?**<br>• **Yes (1)**<br>• **No (0)**	**Did you visit the farmer? If you didn't, why?**	**How did you manage the case?**
1.)			
2.)			

5.2) How does the number of cases seen the last one month, and their management compare with those you saw in September?

- More **(0)**

- Less **(1)**
- Same **(2)**

5.3) Can you list three zoonotic diseases in your area?

-

5.4) How worried are you about you or someone in your family may get an infection with a resistant bacteria?

- Not worried at all **(0)**
- A little bit worried **(1)**
- Very worried **(2)**

5.5) How likely do you think it is that you are exposed to resistant bacteria when you visit a dairy farm?

- Not likely at all **(0)**
- It may happen, but less than 1/3 likelihood **(1)**
- Probably around 50% risk **(2)**
- It is very likely, probably most farms have it **(3)**

5.6) How likely do you think it is that you are exposed to resistant bacteria when you visit a hospital?

- Not likely at all **(0)**
- It may happen, but less than 1/3 likelihood **(1)**
- Probably around 50% risk **(2)**
- It is very likely, probably most hospitals have it **(3)**

5.7) What do you see as the main challenge of tackling AMR in the villages where you are based?
